# Msi2‐mediated MiR7a‐1 processing repression promotes myogenesis

**DOI:** 10.1002/jcsm.12882

**Published:** 2021-12-08

**Authors:** Wenjun Yang, Lele Yang, Jianhua Wang, Yuanyuan Zhang, Sheng Li, Qi Yin, Jinlong Suo, Ruimiao Ma, Yuzhen Ye, Hong Cheng, Jinsong Li, Jingyi Hui, Ping Hu

**Affiliations:** ^1^ Department of Pediatric Orthopedics Xin Hua Hospital Affiliated to Shanghai Jiao Tong University, School of Medicine Shanghai China; ^2^ Guangzhou Laboratory Guangzhou China; ^3^ Max Planck Center for Tissue Stem Cells and Regenerative Medicine Bioland Laboratory Guangzhou China; ^4^ Department of Orthopaedic Surgery Xin Hua Hospital Affiliated to Shanghai Jiao Tong University, School of Medicine Shanghai China; ^5^ State Key Laboratory of Molecular Biology Shanghai Institute of Biochemistry and Cell Biology, Center for Excellence in Molecular Cell Science, Chinese Academy of Sciences Shanghai China; ^6^ Department of Orthopedic Surgery and Institute of Microsurgery on Extremities Shanghai Jiaotong University Affiliated Sixth 's Hospital Shanghai China; ^7^ Shanghai Key Laboratory of Molecular Andrology, State Key Laboratory of Molecular Biology Shanghai Institute of Biochemistry and Cell Biology, Center for Excellence in Molecular Cell Science, Chinese Academy of Sciences Shanghai China; ^8^ School of Life Science and Technology Shanghai Tech University Shanghai China; ^9^ Institute for Stem Cell and Regeneration, Chinese Academy of Sciences Beijing China

**Keywords:** Msi2, HuR, MiR7a‐1 processing, Myogenesis, Skeletal muscle ageing

## Abstract

**Background:**

Most of the microRNAs (MiRs) involved in myogenesis are transcriptional regulated. The role of MiR biogenesis in myogenesis has not been characterized yet. RNA‐binding protein Musashi 2 (Msi2) is considered to be one of the major drivers for oncogenesis and stem cell proliferation. The functions of Msi2 in myogenesis have not been explored yet. We sought to investigate Msi2‐regulated biogenesis of MiRs in myogenesis and muscle stem cell (MuSC) ageing.

**Methods:**

We detected the expression of Msi2 in MuSCs and differentiated myotubes by quantitative reverse transcription PCR (RT‐qPCR) and western blot. Msi2‐binding partner human antigen R (HuR) was identified by immunoprecipitation followed by mass spectrometry analysis. The cooperative binding of Msi2 and HuR on MiR7a‐1 was analysed by RNA immunoprecipitation and electrophoresis mobility shift assays. The inhibition of the processing of *pri‐MiR7a‐1* mediated by Msi2 and HuR was shown by Msi2 and HuR knockdown. Immunofluorescent staining, RT‐qPCR and immunoblotting were used to characterize the function of MiR7a‐1 in myogenesis. Msi2 and HuR up‐regulate cryptochrome circadian regulator 2 (Cry2) via MiR7a‐1 was confirmed by the luciferase assay and western blot. The post‐transcriptional regulatory cascade was further confirmed by RNAi and overexpressing of Msi2 and HuR in MuSCs, and the *in vivo* function was characterized by histopathological and molecular biological methods in Msi2 knockout mice.

**Results:**

We identified a post‐transcription regulatory cascade governed by a pair of RNA‐binding proteins Msi2 and HuR. Msi2 is enriched in differentiated muscle cells and promotes MuSC differentiation despite its pro‐proliferation functions in other cell types. Msi2 works synergistically with another RNA‐binding protein HuR to repress the biogenesis of MiR7a‐1 in an Msi2 dose‐dependent manner to regulate the translation of the key component of the circadian core oscillator complex Cry2. Down‐regulation of Cry2 (0.6‐fold, vs. control, *P* < 0.05) mediated by MiR7a‐1 represses MuSC differentiation. The disruption of this cascade leads to differentiation defects of MuSCs. In aged muscles, Msi2 (0.3‐fold, vs. control, *P* < 0.01) expression declined, and the Cry2 protein level also decreases (0.5‐fold, vs. control, *P* < 0.05), suggesting that the disruption of the Msi2‐mediated post‐transcriptional regulatory cascade could attribute to the declined ability of muscle regeneration in aged skeletal muscle.

**Conclusions:**

Our findings have identified a new post‐transcriptional cascade regulating myogenesis. The cascade is disrupted in skeletal muscle ageing, which leads to declined muscle regeneration ability.

## Introduction

Skeletal muscle has a tremendous capability to regenerate. Muscle regeneration is critical for muscle mass and function maintenance. Muscle stem cells (MuSCs) are the major force that drives postnatal muscle regeneration.^1^ Upon injury, quiescent MuSCs are activated and undergo proliferation and differentiation to form new myofibres and regenerate functional skeletal muscles.^2^ Myogenesis is a highly regulated process orchestrated by many factors.^3^ It is a good paradigm to study the mechanisms regulating adult stem cell proliferation and differentiation. Myogenic regulatory factors and other transcription factors such as Pax7 and Pax3 have been shown to play important roles in orchestrating the muscle regeneration process.^4^, ^5^, ^6^, ^7^ Besides transcription regulation, recent studies have suggested that post‐transcriptional regulation is also essential for skeletal muscle regeneration.^4^, ^8^ Myo‐microRNAs (MiRs), which are MiRNAs specifically expressed in skeletal muscle, are required for MuSC proliferation, differentiation and muscle regeneration by modulating translation of several proteins critical for myogenesis.^9^, ^10^ Many other MiRs have also been demonstrated to be required for muscle regeneration.^11^, ^12^ Most of the MiRs involved in myogenesis are transcriptional regulated. The roles of MiR biogenesis in myogenesis and muscle regeneration have not been fully explored yet.

RNA‐binding proteins bind target RNAs and modulate the fate of RNAs from their synthesis to decay.^13^ Musashi (Msi) protein family, which contains Msi1 and Msi2 in mammals, is a group of highly conserved RNA‐binding proteins regulating alternative pre‐mRNA splicing, mRNA polyadenylation, stabilization, and translation.^14^, ^15^, ^16^, ^17^, ^18^ Msi1 and Msi2 display over 75% amino acid identity and share common tandem RNA recognition motifs, expression patterns and functions.^19^ They are highly enriched in stem cells and tumour cells and considered to be part of the major drivers for oncogenesis and stem cell proliferation.^14^ The functions of Msi proteins in myogenesis have not been explored yet.

Here, we identified a new post‐transcription regulatory cascade governed by two RNA‐binding proteins Msi2 and HuR. Despite the expression pattern in other cell types, Msi2, but not Msi1, is enriched in differentiated muscle cells and required for the differentiation of MuSCs.^15^, ^20^, ^21^ By interacting with HuR, Msi2 functions as a switch to trigger efficient inhibition of MiR7a‐1 biogenesis from *pri‐MiRNA*, which in turn relieves the translation inhibition of circadian regulator Cry2, to allow MuSCs differentiation. Our results suggest that the dosage balance between Msi2 and HuR is lost and the microRNA biosynthesis regulatory cascade is disrupted during ageing, which may lead to defects of MuSC differentiation in old mice. The same mechanism may also apply to human ageing.

## Materials or subjects methods

### Animals

C57BL/6 mice (Charles River, Wilmington, MA, USA) > 24 months were used as the old mouse model. C57BL/6 mice (Charles River) 3 months were used as the young mouse model. The gender of the mice was randomly selected. Animal care and use were in accordance with the guidelines of the Shanghai Institute of Biochemistry and Cell Biology, Chinese Academy of Sciences. Mice within each sample group were selected randomly. In each group, five independent mice were used, and data from all animal samples were collected and subjected for statistical analysis. Msi2 knockout (KO) mice were generated by Cas9‐mediated gene editing.^22^, ^23^ Ten nucleotides were depleted in the first exon of Msi2 gene, and a frame shift was introduced after the 16th amino acid, resulting in the abolishment of Msi2 expression in KO mice. Muscle injury was induced by the injection of cardiotoxin (CTX) (Sigma, San Louise, MO, USA) as previously described to tibialis anterior (TA) muscle.^24^ Briefly, 20 μL of 10 μM CTX was injected at each injection site using 28‐gauge needles. For TA muscle, three injections were usually performed for one piece of muscle. The needle was inserted parallel to the muscle fibre and injected CTX. Two injections were located close to each end of the TA muscle. The third injection was performed at the middle of the TA muscle.

### Gene expression analysis

Total RNA was isolated using RNeasy kits (Qiagen, Germantown, MD, USA). One microgram of total RNA from each sample was reverse transcribed to cDNA using MuLV transcriptase (NEB, Ipswich, MA, USA) and oligo dT primer according to the manufacturer's instruction. Briefly, RNA was first denatured at 85°C for 3 min. MuLV reverse transcriptase was then added and incubated at 42°C for 1 h. Quantitative PCR (qPCR) reactions were performed in triplicates using SYBR Green PCR master mix (DBI, Hazleton, PA, USA) in BioRad thermocycler system (BioRad, Herculase, CA) and analysed by iQ5 optical system software (BioRad). The primers for quantitative reverse transcription PCR (RT‐qPCR) are listed in *Table* 1.

**Table 1:** The primers used for RT‐qPCR

The primers used for RT‐qPCR
Mouse Msi2‐F: TGAGGCAGTGGCTACACAAG
Mouse Msi2‐R: GCTGGTATGGGGTTGAGAGA
Mouse Msi1‐F: AAGAGTGTCTGGTGATGCGG
Mouse Msi1‐R: GGCATCATCCACCTTTCCGA
Mouse HuR‐F: AGAGAGGCAGATTCGCAAGCGT
Mouse HuR‐R: TGCAAAGCTGCAGGGTGACCC
Mouse Cry2‐F: CGGGGTCCGGGTATTTGACG
Mouse Cry2‐R: CTCTCAGGAGTCCTTGCTTGC
Mouse *pri‐MiR7a‐1*‐F: TGCTGGAAGAAGCCTTAACCA
Mouse *pri‐MiR7a‐1*‐R1: TGTCTCTTCTACATTTTCTACAGGC
Mouse *pre‐MiR7a‐1*‐F: GTCAACTGGAAGACTAGTGATTTTGTTG
Mouse *pre‐MiR7a‐1*‐R: CACTCCTATGGCAGACTGTGATTTGT
Mouse uncleaved *pri‐MiR7a‐1*‐R2: TAGAGGTGGCCTGTGCCATA
Mouse mature MiR7a‐1‐F: ACACTCCAGCTGGGTGGAAGACTAGTGAT
Mouse mature MiR7a‐1‐R: CTCAACTGGTGTCGTGGAGTCGGC
Mouse MiR7a‐1‐stem‐loop reverse transcription primer: CTCAACTGGTGTCGTGGAGTCGGCAATTCAGTTGAGACAACAAA
Mouse Hnrnpk F: TTCCCCAACACCGAAACCAA
Mouse Hnrnpk R: TGTCTCTTCTACATTTTCTACAGGC
Mouse MyHC F: ATGCCACCTTCGCTACAACA
Mouse MyHC R: GTTCAGCACTCGGTATCTCTGT
Mouse MCK F: CATGGAGAAGGGAGGCAATA
Mouse MCK R: GACGAAGGCGAGTGAGAATC
Mouse GAPDH‐F: ACCCAGAAGACTGTGGATGG
Mouse GAPDH‐R: ACACATTGGGGGTAGGAACA
Mouse U6‐F: CTTCGGCAGCACATATACTA
Mouse U6‐R: ATATGGAACGCTTCACGAAT
Mouse *pri‐MiR17* F: CTACTGCAGTGAGGGCACTT
Mouse *pri‐MiR17* R: CCGACTGACCGACGACTG
Mouse *pri‐MiR19a* F: TCAAGCAAGCATGTAGGGGT
Mouse *pri‐MiR19a* R: ATAGCAGGCCACCATCAGTT
Mouse *pri‐MiR23a* F: TTAGGTGCTACACTCCGCTC
Mouse *pri‐MiR23a* R: CAGAGCACAGGGTCAGTTGG
Mouse *pri‐MiR27a* F: GTCGCCAAGGATGTCTGTCT
Mouse *pri‐MiR27a* R: AGCCACTGTGAACACGACTT
Mouse *pri‐MiR23b* F: CAGCACATGTGGATGGGAGT
Mouse *pri‐MiR23b* R: AGGTCATGGTTGCGTGGTAA
Mouse *pri‐MiR27b* F: CCGGCGATGACCTCTCTAAC
Mouse *pri‐MiR27b* R: CTCCTCCTCTGGAGTGACCA
Mouse *pri‐MiR125a* F: TCCACCATAGCTACACTGCC
Mouse *pri‐MiR125a* R: GCCAGGGGTCACCTGAAATC
Mouse *pri‐let7b* F: GATGTTGCCCCTCCGAAGAT
Mouse *pri‐let7b* R: GTCATAGCCCCCACCCAATC
Mouse *pri‐let7i* F: GTGGAGATAACTGCGCAAGC
Mouse *pri‐let7i* R: GCGCCCCGGAAAACAAAG
Mouse *pri‐MiR128‐1* F: AGTGACGTTAGCAATGACAGGT
Mouse *pri‐MiR128‐1* R: AGCACATAAGGAGAAGCTGCAA

RT‐qPCR, quantitative reverse transcription PCR.

### Cell culture and differentiation

Primary MuSCs were isolated as previously described.^24^ Briefly, TA muscles from 3‐month‐old mice were dissected and dissociated with collagenase (Roche, Indianapolis, IN, USA). The cells were negatively selected by biotinylated CD45, CD11, CD31 and Sca1 antibodies. The muscle cells in the flowthrough were subjected to CD34‐FITC (BD biosciences) and integrin α_7_‐allophycocyanin (R&D systems, Minneapolis, MN, USA) staining. The viable PI^−^CD34^+^integrin‐α_7_^+^ MuSCs were collected by FACS sorting (Influx, BD biosciences, Franklin Lake, NJ, USA). MuSCs were cultured on collagen coated dishes in F10 medium containing 10% foetal bovine serum (FBS), 5 ng/mL IL‐1α, 5 ng/mL IL‐13, 10 ng/mL IFN‐γ, and 10 ng/mL TNF‐α (R&D Systems), and 2.5 ng/mL FGF (Invitrogen, Red Wood City, CA, USA) as described previously.^24^ MuSCs were differentiated in differentiation medium [DMEM medium (invitrogen)] containing 2% horse serum (HyClone, Malborough, MA, USA) for 3 days. C2C12 cells (ATCC) were cultured in DMEM medium (Invitrogen) containing 10% FBS (HyClone) and differentiated in DMEM medium (Invitrogen) containing 2% horse serum (HyClone) for 3 days. The differentiated myotubes were further isolated by pre‐attaching to plates for three times.

### microRNA product detection

For mature MiRs, stem‐loop reverse transcription primer was used for reverse transcription. MiR7a‐1‐F and MiR7a‐1‐R primers were used for quantitative PCR to detect 23 nt mature MiR7a‐1.

### Virus injection

Adenovirus encoding MiR7a‐1 or scramble control was injected into the pre‐injured TA muscles of 3‐month‐old C57BL/6 (Charles River) male mice. Fifty microlitres of adenovirus encoding MiR7a‐1 (1.5 × 10^7^ unit/μL) or scramble (1.5 × 10^7^ unit/μL) was injected into TA muscle once a day for five continuous days. The first injection was together with CTX injection. TA muscles were harvested 2 days after the last injection (7 days after CTX injection). For each type of virus, five mice were injected.

### Immunofluorescent staining

For immunofluorescent staining, muscle sections were fixed with 4% parafamaldehyde, permeabilized with 0.5% Triton X‐100 for 15 min and blocked for 1 h at room temperature by goat serum (HyClone). Primary antibodies were incubated at room temperature for 2 h. The cells and sections were next stained with Alexa 488‐labelled, 561‐labelled or 647‐labelled anti‐mouse or anti‐rabbit antibodies (Invitrogen). All images were acquired by confocal microscopy (Leica, Wetzlar, Germany).

### Antibodies

Immunofluorescent stainings for muscle sections and myotubes and immunoblottings were performed with the following antibodies: Rabbit anti‐Msi2 (Millipore, 04‐1069), Mouse anti‐Msi2 (Abnova, H00124540‐M11), Rabbit anti‐HuR (Cell Signaling Technology, 12582), Mouse anti‐HuR (Santa Cruz, sc‐5261), Mouse anti‐Flag tag (Abmart, ab1162), Rabbit anti‐Myc tag (Proteintech, 16286‐1‐AP), Rabbit anti‐Laminin (Abcam, ab11575), Mouse anti‐MyHC (Upstate, 05‐715), Mouse anti‐GAPDH (Protein tech, 60004), Rabbit IgG (Santa Cruz, sc‐2027), Mouse IgG (Santa Cruz, sc‐2025), Rabbit anti c‐Myc agarose beads (Sigma, A7470) and Mouse anti‐Flag agarose beads (Sigma, A2220).

### RNA immunoprecipitation‐quantitative PCR assay

Muscle stem cells transfected with Flag‐tagged Msi2 or HuR were UV crosslinked in an UV box (Stratalinker 2400, Stratagene) at 200 mJ/cm^2^, 254 nm and harvested cells in PBS. Cell pellets were lysed in lysis buffer containing 50 mM Tris–HCl, pH 7.4, 100 mM NaCl, 1% NP‐40, 0.1% SDS, 0.5% sodium deoxycholate and protease inhibitors (Roche). Antibody coupled agarose beads or Dybeads were applied to the lysate and immunoprecipitation (IP) was performed. The precipitated RNA was isolated by Trizol (Sigma) and subjected for reverse transcription with reverse transcriptase III (Invitrogen). qpCR reactions were performed using the RT products as templates.

### RNA electrophoretic mobility shift assay

Cy3‐labelled RNA probes [wild‐type (WT) probe: UAAUUCGUACUUUUUUUUUCUUUUCUUUUAUAGUGUGAA; mutant probe: UAAUUCGUACTTGCTTCCTCTTTACTTGTAUAGUGUGAA] were synthesis by Shanghai Generay Biotech Company. Recombinant Msi2, HuR, or the combination of Msi2 and HuR were incubated with Cy3‐labelled RNA in binding buffer containing 100 mM Tris–HCl, pH 7.5, 7.5 mM KCl, 25 mM MgCl_2_, 375 mM NaCl, 12.5% glycerol and 5 mM DTT at 30°C for 30 min, respectively. RNA‐protein complexes were fractionated by a 6% non‐denaturing polyacrylamide gel at 120 V for 35 min, at 4°C, and visualized with a FUJIFILM FLA9000 image scanner.

### Myofibre diameter and cross‐sectional area measurement

Myofibre diameter was measured by Adobe Acrobat 9 pro software (Adobe). Three independent visual fields in each sample were chosen randomly. Three fibres from each visual field were measured for each treatment. The myofibre cross‐sectional area was measured by Image J software (National Institutes of Health). Three independent visual fields in each sample were chosen randomly. Three myofibres from each visual field were measured for analysis. The identity of the samples was blinded to the personnel who performed the measurement.

### Statistical analysis

Two‐tailed Student's test was performed for pairwise comparison among groups. Statistical significance was set at *P* < 0.05.

## Results

### Msi2 expression is up‐regulated during muscle stem cell differentiation

**Figure 1:**
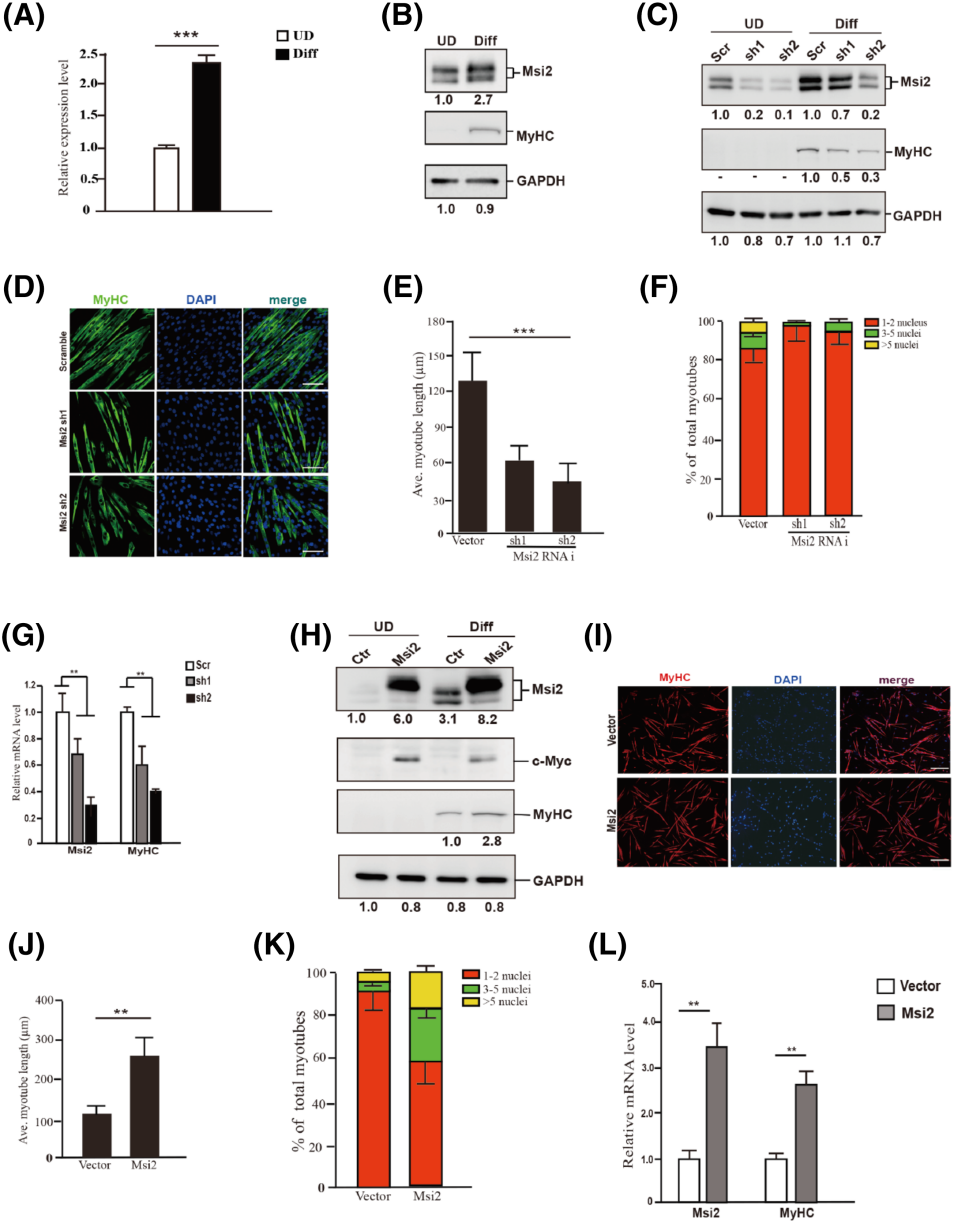
Msi2 expression increased in differentiated MuSCs and required for MuSC differentiation. (*A*) Expression levels of Msi2 in primary MuSCs before and after differentiation, respectively. RT‐qPCR was performed with RNA extracted from the primary MuSCs before or after differentiation. The results were normalized to GAPDH. Error bars indicated standard deviation and were based on three independent experiments. *** indicated *P* < 0.001. (*B*) Protein levels of Msi2 in primary MuSCs before and after differentiation, respectively. Immunoblotting assays were performed with whole cell extracts from primary MuSCs or myotubes differentiated from MuSCs. MyHC indicated the differentiation status of MuSCs. GAPDH served as an internal control. The numbers below each panel indicated relative signal intensity. (*C*) Protein levels of Msi2 in undifferentiated and differentiated primary MuSCs treated by control or two pieces of Msi2‐specific shRNAs. Immunoblotting assays were performed with whole cell extracts. GAPDH served as an internal control. The numbers below each panel indicated relative signal intensity. (*D*) Immunofluorescent staining of MyHC in myotubes differentiated from MuSCs treated with scramble or two Msi2‐specific shRNAs. Green indicated MyHC; blue indicated DAPI staining of nuclei; merge indicated the merged images of green and blue. Scale bars: 50 μm. (*E*) Statistical analysis of the average length of myotubes. Error bars indicated standard deviation calculated based on four independent experiments. *** indicated *P* < 0.001. (*F*) Statistical analysis of the fusion index of myotubes. The number of nuclei in each myotube was counted and analysed. Five hundred myotubes were counted in each sample. (*G*) Expression levels of Msi2 and MyHC in MuSCs described in (*D*). RT‐qPCR assays were performed with RNA extracted from differentiated MuSCs. Error bars indicated standard deviation and were based on three independent experiments. ** indicated *P* < 0.01. (*H*) Immunoblotting of Msi2 in MuSCs before and after differentiation. MuSCs were infected with adenovirus encoding Myc‐tagged Msi2 or vector. The whole cell protein extracts from the undifferentiated MuSCs or differentiated myotubes were subjected for immunoblotting assays using antibodies against Msi2, MyHC, Myc and GAPDH. GAPDH served as an internal control. The numbers below each panel indicated relative signal intensity. (*I*) Immunofluorescent staining of MyHC in myotubes overexpressing Msi2. MuSCs were infected with adenovirus encoding Msi2 followed by differentiation. Red indicated MyHC; blue indicated DAPI staining of nuclei; merge indicated the merged images of red and blue. Scale bars: 100 μm. (*J*) Statistical analysis of the average length of myotubes. Error bars indicated standard deviation calculated based on four independent experiments and ** indicated *P* < 0.01. (*K*) Statistical analysis of the fusion index of myotubes. The number of nuclei in each myotube was counted and analysed. Five hundred myotubes were counted in each sample. Expression levels of Msi2 and MyHC in MuSCs described in (*I*). (*L*) RT‐qPCR assays were performed with RNA extracted from differentiated myotubes. Error bars indicated standard deviation and were based on three independent experiments. ** indicated *P* < 0.01. Msi2, Musashi 2; MuSCs, muscle stem cells; MyHC, Myosin heavy chain; RT‐qPCR, quantitative reverse transcription PCR.

When we surveyed the expression levels of RNA‐binding proteins in MuSCs and differentiated myotubes, to our surprise, Msi2, a well‐known factor to promote stem cell proliferation is up‐regulated in differentiated myotubes at both mRNA and protein level (*Figure* 1A and 1B). In contrast, Msi1 was mainly expressed in MuSCs, and its expression decreased in differentiated myotubes (Supporting Information, *Figure* S1), which is consistent with the previous findings.^14^ We therefore went on to investigate the functions of Msi2 during myogenesis.

Knocking down Msi2 by RNAi led to differentiation defects in MuSCs as indicated by the decreased frequency of myotube formation and shorter myotubes (*Figure* 1D–1F). Consistent with the morphological changes, the expression level of differentiation marker Myosin heavy chain (MyHC) was down‐regulated after Msi2 RNAi (*Figure* 1C and 1G). We next overexpressed Myc‐tagged Msi2 in MuSCs (*Figure* 1H and 1L) and observed improved differentiation as indicated by longer myotubes and higher fusion index (*Figure* 1I–1K). The expression levels of MyHC were also increased (*Figure* 1H and 1L). Taken together, these results suggest that Msi2 is required for MuSC differentiation, which is opposite to the pro‐proliferation functions of Msi2 in other stem cell types.

### Msi2 and HuR repress the biogenesis of MiR7a‐1

**Figure 2:**
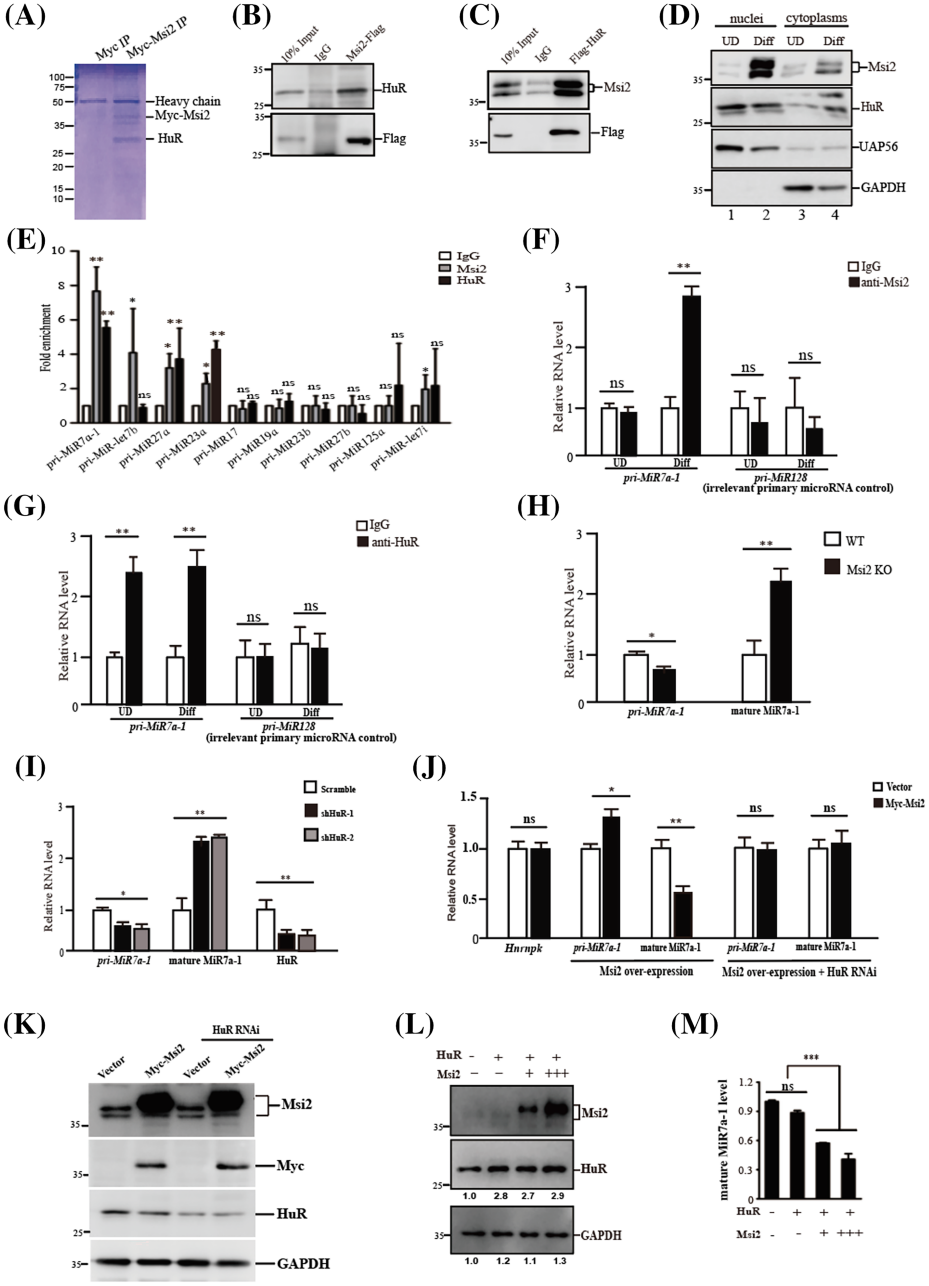
Msi2 and HuR inhibited the biogenesis of MiR7a‐1. (*A*) Coomassie blue staining of Msi2 immunoprecipitation. C2C12 cells were infected by adenovirus encoding Myc‐tagged Msi2 and differentiated. Whole cell protein extracts from differentiated myotubes were subjected for immunoprecipitation using anti‐Myc antibody. The immunoprecipitated proteins were subjected for SDS‐PAGE analysis followed by Coomassie staining, and each band was identified by mass spectrometry. The protein names were labelled according to mass spectrometry results. (*B*) Immunoprecipitation followed by immunoblotting to confirm the protein–protein interaction between Msi2 and HuR. MuSCs were infected by adenovirus encoding Flag‐tagged Msi2. Whole cell protein extracts from the infected cells were subjected to anti‐Flag immunoprecipitation followed by HuR immunoblotting. IgG immunoprecipitation serves as the control. (*C*) Immunoprecipitation followed by immunoblotting to confirm the protein–protein interaction between Msi2 and HuR. MuSCs were infected by adenovirus encoding Flag‐tagged HuR. Whole cell protein extracts from the infected cells were subjected to anti‐Flag immunoprecipitation followed by Msi2 immunoblotting. IgG immunoprecipitation serves as the control. (*D*) Subcellular distribution of Msi2 and HuR. Undifferentiated and differentiated MuSCs were harvested, and nuclear and cytoplasmic proteins were fractionated, respectively. Immunoblottings of Msi2, HuR, UAP56 and GAPDH were performed. UAP56 was localized in nuclei and served as a control for proper isolation of nuclei. GAPDH was a cytoplasmic localized protein and served as a control for the proper isolation of cytoplasm. (*E*) RIP PCR analysis using Msi2 or HuR antibody. RIP PCR assays were performed using MuSCs ectopically expressing Myc‐tagged Msi2 and Flag‐tagged HuR. Error bars indicated standard deviation and were based on three independent experiments. * indicated *P* < 0.05. ** indicated *P* < 0.01. ns indicated no significant changes. (*F*) RIP PCR analysis using anti‐Msi2. MuSCs and differentiated myotubes were harvested for RIP assays. *Pri‐MiR7a‐1* and *pri‐MiR128* were detected by RT‐qPCR. *Pri‐MiR128* was an irrelative MiRNA serving as a negative control. Error bars indicated standard deviation and were based on three independent experiments. ** indicated *P* < 0.01. ns indicated no significant changes. (*G*) RIP PCR analysis using anti‐HuR. MuSCs and differentiated myotubes were harvested for RIP assays. *Pri‐MiR7a‐1* and *pri‐MiR128* were detected by RT‐qPCR. *Pri‐MiR128* was an irrelative MiRNA serving as a negative control. Error bars indicated standard deviation and were based on three independent experiments. ** indicated *P* < 0.01. ns indicated no significant changes. (*H*) The expression level of *pri‐MiR7a‐1* and mature MiR7a‐1 in WT and Msi2 knockout muscle cells. MuSCs were isolated from WT and Msi2 knockout mice followed by differentiation. RT‐qPCR assays were performed using total RNAs extracted from the differentiated cells. Error bars indicated standard deviation and were based on three independent experiments. * indicated *P* < 0.05. ** indicated *P* < 0.01. (*I*) The expression levels of *pri‐MiR7a‐1*, mature MiR7a‐1 and HuR in differentiated MuSCs treated with shRNA against HuR. MuSCs were infected by adenovirus encoding shRNA against HuR and differentiated for 3 days. Two pieces of shRNAs were used. The expression levels of *pri‐MiR7a‐1*, MiR7a‐1 and HuR were detected by RT‐qPCR. Error bars indicated standard deviation and were based on three independent experiments. * indicated *P* < 0.05. ** indicated *P* < 0.01. (*J*) The expression levels of *Hnrnpk*, *pri‐MiR7a‐1* and mature MiR7a‐1 in MuSCs ectopically expressing Myc‐tagged Msi2. RT‐qPCR assays were performed to detect the expression levels of MiR7a‐1 host gene *Hnrnpk*, *pri‐MiR7a‐1* and mature MiR7a‐1. ShRNA against HuR was further introduced to MuSCs overexpressing Msi2. The expression levels of *pri‐MiR7a‐1* and mature MiR7a‐1 were examined by RT‐qPCR assays. Error bars indicated standard deviation and were based on three independent experiments. * indicated *P* < 0.05. ** indicated *P* < 0.01. ns indicated no significant changes. (*K*) The protein levels of Msi2 and HuR in MuSCs ectopically expressing Myc‐tagged Msi2. Immunoblottings were performed to detect the protein level of Msi2 and HuR. ShRNA against HuR was further introduced to MuSCs overexpressing Msi2. The protein levels of Msi2 and HuR were detected by immunoblotting. GAPDH served as the internal control. (*L*) The protein levels of Msi2 and HuR in MuSCs overexpressing Msi2. MuSCs were infected by increasing amounts of adenovirus encoding Myc‐tagged Msi2 and the same amount of Flag‐tagged HuR. Whole cell extracts proteins were subjected for immunoblotting with anti‐Msi2, anti‐HuR and anti‐GAPDH antibodies. GAPDH served as an internal control. (*M*) The relative level of mature MiR7a‐1. MuSCs were infected by increasing amounts of adenovirus encoding Myc‐tagged Msi2 and the same amount of Flag‐tagged HuR. The level of mature MiR7a‐1 was examined by RT‐qPCR. Error bars indicated standard deviation and were based on three independent experiments. *** indicated *P* < 0.001. ns indicated no significant changes. HuR, human antigen R; Msi2, Musashi 2; MuSCs, muscle stem cells; RT‐qPCR, quantitative reverse transcription PCR; RIP, RNA immunoprecipitation; WT, wild type.

We next set out to investigate the mechanism of the functions of Msi2 by identifying the interaction proteins of Msi2 in muscle cells. Myc‐tagged Msi2 was expressed in C2C12 myoblasts, and anti‐Myc IP was performed. The immunoprecipitated materials were subjected to SDS‐PAGE electrophoresis followed by mass spectrometry (MS) analysis (*Figure* 2A). MS analysis revealed that HuR is the major partner of Msi2 (*Table* 2). To confirm the MS results, we performed IP assays coupled with immunoblotting analysis. Anti‐Flag IP was performed using MuSCs expressing Flag‐tagged Msi2, and HuR protein was detected by anti‐HuR immunoblotting. Msi2 was able to immunoprecipitate HuR (*Figure* 2B). Vice versa, Flag‐tagged HuR was also able to immunoprecipitate Msi2 in MuSCs (*Figure* 2C), and the interaction is RNA independent (*Figure* S2F–S2H). Together, these results indicate that Msi2 and HuR interact with each other in muscle cells in an RNA‐independent manner.

**Table 2:** Proteins identified by mass spectrometry

#	Description	Coverage	# of peptides
1	ELAV‐like protein 1 (HuR)	49.39	19
2	28S ribosomal protein S22, mitochondrial	47.63	23
3	RNA‐binding protein Musashi homologue 2 (Msi2)	44.51	23
4	Insulin‐like growth factor 2 mRNA‐binding protein 2	41.72	25
5	heterogeneous nuclear ribonucleoproteins C1/C2 isoform 2	40.33	18
6	28S ribosomal protein S27, mitochondrial	36.87	17
7	28S ribosomal protein S29, mitochondrial isoform 2	29.55	13
8	Nucleolin	26.03	22
9	Putitative helicase MOV‐10 isoform b	24.8	22
10	Myelin expression factor 2 isoform 1	24.7	16

HuR RNAi resulted in repressed MuSC differentiation (*Figure* S2A–S2D), which is consistent with the previous observations.^25^ These results suggest that HuR is also required for myogenesis.

We further checked the subcellular localization of Msi2 and HuR in MuSCs and differentiated myotubes. Whole cell extracts of MuSCs and myotubes were fractionated to nuclear and cytoplasmic fractions as described previously.^26^, ^27^ Immunoblotting assays were performed. Msi2 was detected in both nuclear and cytoplasmic fractions in MuSCs before differentiation (*Figure* 2D, lanes 1 and 3). After differentiation, not only the total protein level of Msi2 but also the nuclei located Msi2 increased (*Figure* 1B and 2D, lanes 2 and 4, *Figure* S2E). In contrast, HuR was mainly located in nuclei regardless of the differentiation status (*Figure* 2D).

The nuclei located Msi2 has been proposed to be involved in processing of primary MiR7a‐1 in HeLa cells with HuR.^28^ Whether that is true in other cell types is not known yet. We analysed all the currently available CLIP‐seq data of Msi2 and HuR from all cell types in the database and selected the *pri‐MiR* co‐occupied by both Msi2 and HuR. The candidates were further selected based on their expression levels in C2C12 cells from database. We identified 10 candidates from the pool of primary MiRs bound by both Msi2 and HuR (*Figure* S3A). We further overexpressed Msi2 and HuR and performed RNA immunoprecipitation assays to confirm the binding of both Msi2 and HuR on these primary MiRNAs in MuSCs. Msi2 and HuR co‐bound 3 out of the 10 primary MiRNAs, namely, *pri‐MiR7‐1*, *pri‐MiR27a* and *pri‐MiR23a* (*Figure* 2E).

Among them, Msi2 and HuR displayed the highest affinity to *pri‐MiR7a‐1* (*Figure* 2E). We therefore use MiR7a‐1 as a paradigm to explore the functions of Msi2 and HuR. There are three *pri*‐*MiR7* sequences existing in the genome. Semi‐quantitative RT‐PCR results revealed that *pri‐MiR7a‐2* and *pri‐MiR7b* were hardly detected in MuSCs before and after differentiation, while *pri‐MiR7a‐1* was detected easily under the same condition (*Figure* S3B), suggesting that MiR7a‐1 originated from the last intron of *Hnrnpk* gene is the major precursor of MiR7 in muscle cells.

RNA immunoprecipitation assays were performed to detect the binding of Msi2 and HuR on *pri‐MiR7a‐1* in undifferentiated MuSCs and differentiated myotubes. Msi2 bound *pri‐MiR7a‐1* in differentiated myotubes but not in MuSCs (*Figure* 2F). These results were consistent with the increased Msi2 expression in myotubes (*Figure* 1B). HuR bound *pri‐MiR7a‐1* in both undifferentiated MuSCs and differentiated myotubes (*Figure* 2G). In contrast, neither Msi2 nor HuR bound a control *pri‐MiRNA* (*pri‐MiR128*) in MuSCs or myotubes (*Figure* 2F and 2G). HuR recruitment to *pri‐MiR7a‐1* was abolished in Msi2 KO MuSCs (*Figure* S3C). Conversely, Msi2 binding on *pri‐MiR7a‐1* was abolished in HuR RNAi cells (*Figure* S3D). These results the cooperative binding of Msi2 and HuR on *pri‐MiR7a‐1* in muscle cells.

We next examined whether the binding of *pri‐MiR7a‐1* by Msi2 and HuR has any effects on the level of mature MiR7a‐1. The expression profiles of *pri‐MiR7a‐1*, mature MiR7a‐1, and *Hnrnpk*, the host gene of MiR7a‐1, in undifferentiated MuSCs and differentiated myotubes were measured by RT‐qPCR. The level of *Hnrnpk* transcript did not change after differentiation (*Figure* S3E). The level of *pri‐MiR7a‐1* increased, while the level of mature MiR7a‐1 decreased after differentiation (*Figure* S3F), hinting a potential regulatory step at post‐transcriptional level.

We then analysed the level of primary and mature MiR7a‐1 in Msi2 KO MuSCs. The *pri‐MiR7a‐1* level slightly decreased, while the level of mature MiR7a‐1 increased significantly in Msi2 KO myotubes (*Figure* 2H) suggesting that Msi2 inhibits the processing of *pri‐MiR7a‐1*, but not a control MiRNA (MiR128) (*Figure* S3G).

RNAi against HuR in MuSCs led to slightly decreased *pri‐MiR7a‐1* and significantly increased mature MiR7a‐1 (*Figure* 2I), suggesting that HuR inhibits the processing of MiR7a‐1. Taken together, the above results suggest that both Msi2 and HuR are necessary to inhibit the processing of MiR7a‐1.

After differentiation, Msi2 expression increased, and the mature MiR7a‐1 level decreased (*Figure* 1A, 1B and S3F). The reverse correlation between Msi2 and mature MiR7a‐1 is consistent with the notion that Msi2 inhibits MiR7a‐1 maturation during myogenesis. In contrast, the level of HuR was largely unchanged in nuclei during myogenesis (*Figure* 2D), leading us having the hypothesis that the dosage of Msi2 is key to trigger the inhibition of MiR 7a biogenesis. We next tested this hypothesis. When Msi2 was overexpressed in MuSCs, the level of mature MiR7a‐1 decreased significantly (*Figure* 2J and 2K). Increasing amount of Msi2 leads to more efficient inhibition of MiR7a‐1 processing specifically (*Figure* 2L and 2M & S4A and S4B). These results suggest that the inhibition efficiency depends on the dosage of Msi2. Though HuR was required for efficient inhibition of MiR7a‐1 processing (*Figure* 2I), higher amount of HuR did not lead to further inhibition of MiR7a‐1 processing (*Figure* S4C and S4D). Overexpressing HuR did not rescue the lost repression of MiR7a‐1 procession in Msi2 KO MuSCs, suggesting that HuR alone is not sufficient to efficiently repress the processing of MiR7a‐1 (*Figure* S4E). Increasing amount of Msi2 could not rescue the loss of HuR (*Figure* 2J). These results suggest that HuR and Msi2 are all required for efficient inhibition of MiR7a‐1 processing in muscle cells.

Altogether, these results suggest that HuR provides a basic platform and Msi2 serves as the triggering factor to initiate the efficient inhibition of MiR7a‐1 processing in a dose‐dependent manner.

### Msi2 and HuR bind the consensus HuR recognition site at the flanking region of *pri‐MiR7a‐1*

**Figure 3:**
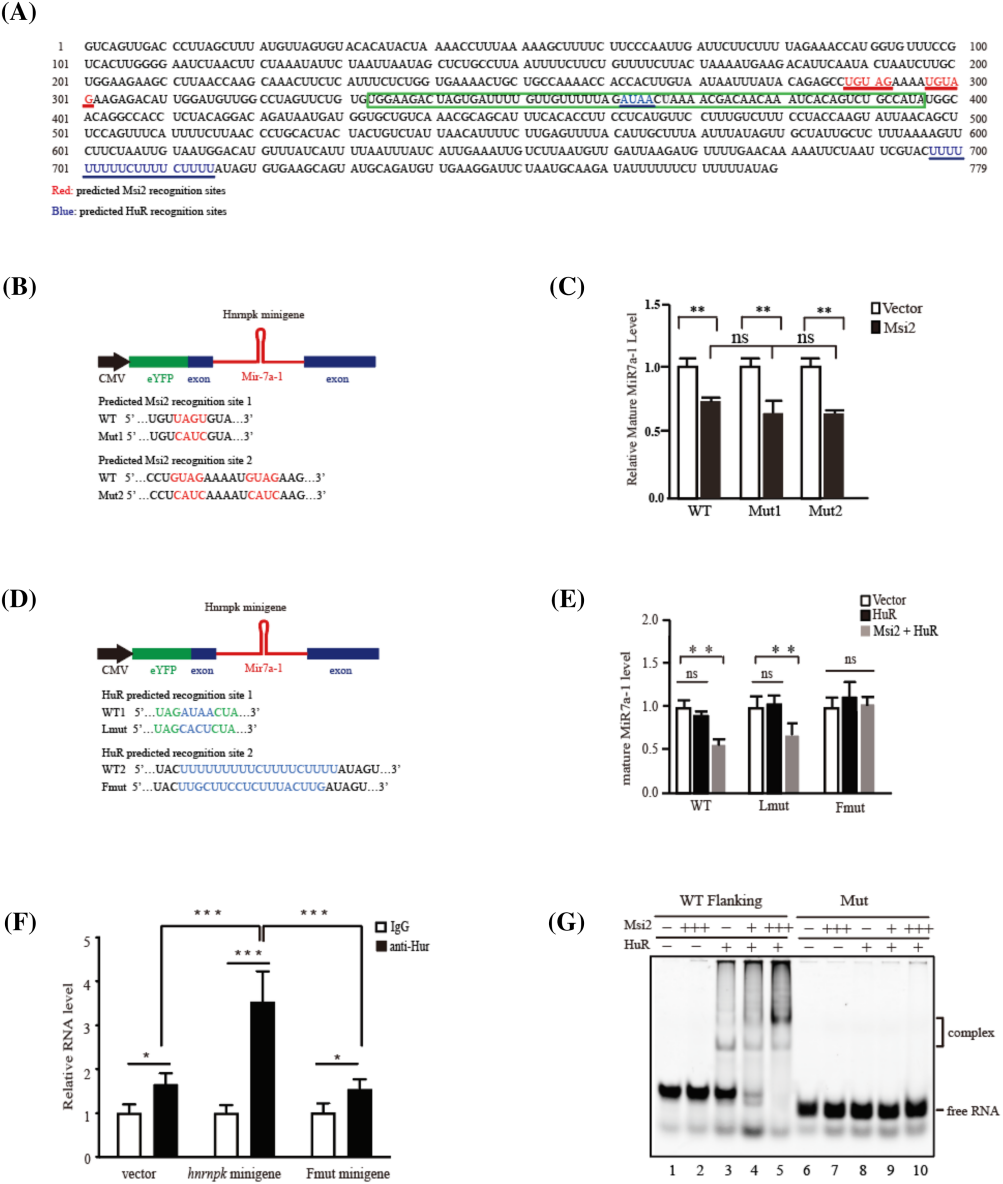
Msi2 and HuR bind the consensus HuR recognition site at the flanking region of *pri‐MiR7a‐*1. (*A*) The sequence of *Hnrnpk* minigene. Red indicted the predicted Msi2 recognition sites. Blue indicated the predicted HuR recognition sites. Green box indicated the sequence of *pre‐MiR7a‐*1. (*B*) The scheme of the *Mir7a‐1* minigene with intact or mutated potential Msi2 recognition sites. The predicted Msi2 recognition sites and the mutated sequences were listed below the schematic graph. Red indicated the predicted Msi2 recognition sites. (*C*) The expression level of mature MiR7a‐1 with intact Msi2 recognition sites or mutated Msi2 recognition sites. Msi2 and *Mir7a‐1* minigene containing WT or mutated Msi2 recognition sites were co‐transfected to MuSCs. RT‐qPCR assays were performed to determine the level of mature MiR7a‐1. Error bars indicated standard deviation and were based on three independent experiments. ** indicated *P* < 0.01. ns indicated no significant changes. (*D*) The scheme of the *Mir7a‐1* minigene with intact or mutated potential HuR recognition sites. The predicted HuR recognition sites and the mutated sequences were listed below the schematic graph. Blue indicated the predicted Msi2 recognition sites. Green indicated the sequence of mature MiR7a‐1. (*E*) The expression level of mature MiR7a‐1 with intact or mutated HuR recognition sites. HuR and *Mir7a‐1* minigene containing WT or mutated HuR recognition sites were co‐transfected to MuSCs. RT‐qPCR assays were performed to determine the level of mature MiR7a‐1. Msi2, HuR and *Mir7a‐1* minigene containing WT or mutated HuR recognition sites were co‐transfected to MuSCs. Error bars indicated standard deviation and were based on 3 independent experiments. ** indicated *P* < 0.01. ns indicated no significant changes. (*F*) Vector, *Mir7a‐1* minigene or mutated *Mir7a‐1* minigene were transfected to primary MuSCs, respectively. RIP PCR assays with anti‐HuR antibody were performed. * indicated *P* < 0.05. *** indicated *P* < 0.001. (*G*) HuR and Msi2 RNA EMSA assays. Synthesized RNA probe containing potential HuR‐binding site was labelled by Cy3. Recombinant Msi2 and HuR purified from HEK293T cells were utilized for electrophoretic mobility shift assay EMSA assays. WT Flanking indicated RNA probe containing the potential HuR recognition site at the flanking region of *pri‐MiR7a‐1*. HuR, human antigen R; Msi2, Musashi 2; MuSCs, muscle stem cells; RT‐qPCR, quantitative reverse transcription PCR; RIP, RNA immunoprecipitation; WT, wild type.

We next analysed the sequence of *pri‐MiR7a‐1* and found two consensus Msi2 binding sites at the loop region and a HuR consensus binding site at the flanking region (*Figure* 3A). To identify the functional binding sites, we constructed a plasmid containing the *Hnrnpk* minigene driven by CMV promoter, which can be transcribed to *pri‐MiR7a‐1* once transfected to cells (*Figure* 3B and 3D). Each of the predicted Msi2 recognition site in the *Hnrnpk* minigene was then mutated (*Figure* 3B). The minigene containing the mutant sites were co‐transfected with plasmid encoding Msi2 to MuSCs, and the mature mouse MiR7a‐1 level was examined.

To our surprise, none of the two mutations relieved the repression of MiR7a‐1 maturation mediated by Msi2 (*Figure* 3C), suggesting that neither of the predicted Msi2 recognition site is required for the repression of MiR7a‐1 maturation.

Mutation of the predicted HuR recognition sites at the flanking region of *pri‐MiR7a‐1* resulted in significant relieving of the MiR7a‐1 maturation inhibition, while mutation at the loop region did not show the similar results (*Figure* 3E). These data suggest that HuR recognition site at the flanking region is important for the inhibition of *pri‐MiR7a‐1* processing in muscle cells.

To further confirm that HuR indeed binds to the flanking region of *pri‐MiR7a‐1* in MuSCs, we transfected *Mir7a‐1* minigene to MuSCs and performed HuR RNA immunoprecipitation. When the minigene was transfected, compared with the vector control, higher level of HuR recruitment was detected. When the predicted HuR‐binding site at the flanking region was mutated, the recruitment of HuR was reduced to the level similar to that in the vector control (*Figure* 3F). These results further confirm the binding of HuR to *pri‐MiR7a‐1* in MuSCs.

We then performed electrophoretic mobility shift assays with RNA probes to confirm Msi2 and HuR binding on the HuR recognition site. HuR alone was able to bind the WT probe (*Figure* 3G, lane 3). When both HuR and Msi2 were present, a strong cooperative binding was observed (*Figure* 3G, lanes 4–5), suggesting that Msi2 and HuR work cooperatively to enhance the formation of protein‐RNA complex. When the HuR‐binding site at the flanking region was mutated, the binding of HuR and the cooperative binding of HuR and Msi2 were all abolished (*Figure* 3G, lanes 6–10). These results suggest that Msi2 enhances HuR binding at the flanking site of *pri‐MiR7a‐1*.

### Msi2 and HuR up‐regulate Cry2 via MiR7a‐1 to promote myogenesis

**Figure 4:**
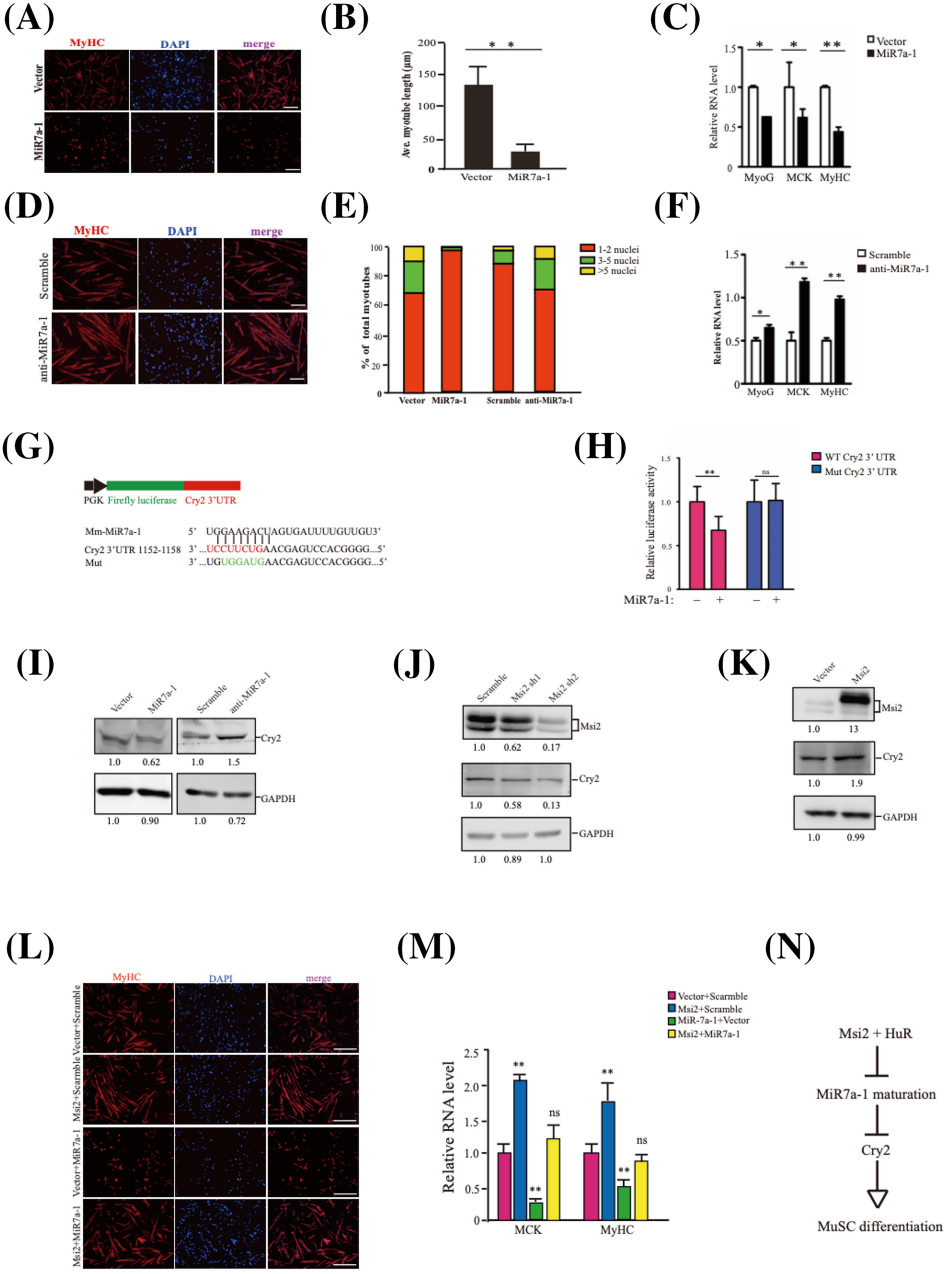
Msi2 and HuR up‐regulate Cry2 via MiR7a‐1 to promote myogenesis. (*A*) Immunofluorescent staining of MyHC in MuSCs overexpressing MiR7a‐1. MuSCs were infected by adenovirus encoding MiR7a‐1 and differentiated. The differentiated MuSCs were stained with anti‐MyHC. Red indicated MyHC; DAPI indicated nuclear staining; merge indicated the merged images of red and blue. Scale bars: 100 μm. (*B*) Statistics of the average length of myotubes overexpressing MiR7a‐1. Error bars indicated standard deviation and were based on three independent experiments. ** indicated *P* < 0.01. (*C*) The expression level of MyoG, MCK and MyHC in differentiated MuSCs overexpressing MiR7a‐1. RT‐qPCR assays were performed with RNA extracted from differentiated MuSCs. Error bars indicated standard deviation and were based on three independent experiments. * indicated *P* < 0.05. ** indicated *P* < 0.01. (*D*) Immunofluorescent staining of MyHC in MuSCs expressing MiR7a‐1 antagomir. MuSCs were infected by MiR7a‐1 antagomir and differentiated. The differentiated MuSCs were stained with anti‐MyHC. Red indicated MyHC; DAPI indicated nuclear staining; merge indicated the merged images of red and blue. Scale bars: 100 μm. (*E*) Statistical analysis of the fusion index of myotubes. The number of nuclei in each myotube was counted and analysed. Five hundred myotubes were counted in each sample. (*F*) The expression level of MyoG, MCK and MyHC in differentiated MuSCs expressing MiR7a‐1 antagomir. RT‐qPCR assays were performed with RNA extracted from differentiated MuSCs. Error bars indicated standard deviation and were based on three independent experiments. * indicated *P* < 0.05. ** indicated *P* < 0.01. (*G*) Scheme of luciferase assay construct for Cry2 3′ UTR. The sequences of MiR7a‐1 complementary to the 3′ UTR of the target gene are in red colour; the mutated sequences are in green colour. (*H*) The luciferase activity of reporter gene carrying MiR7a‐1 target sequence from Cry2 at the 3′ UTR region. Firefly luciferase activity regulated by MiR7a‐1 target sequence at the 3′ UTR. Renilla luciferase activity driven by CMV promoter served as an internal control. Error bars indicated standard deviation and were based on five independent experiments. ** indicated *P* < 0.01. ns indicated no significant changes. (*I*) Protein levels of Cry2 after MiR7a‐1 or MiR7a‐1 antagomir overexpression. MuSCs transfected with MiR7a‐1 or MiR7a‐1 antagomir were harvested, and the whole cell protein extracts were subjected foranti‐Cry 2 immunoblotting. GAPDH served as an internal control. Numbers under each lane indicated signal intensity. (*J*) The protein levels of Cry2 in Msi2 RNAi MuSCs. MuSCs were infected by adenovirus encoding shRNAs against Msi2 or scramble RNA and differentiated for 3 days. Two pieces of shRNAs were used. Whole cell protein extracts were subjected for immunoblottings using anti‐Msi2 and Cry2. GAPDH served as an internal control. The numbers below each lane indicated the signal intensity. (*K*) The protein levels of Cry2 in Msi2 overexpressing MuSCs. MuSCs were infected by adenovirus encoding Msi2. Whole cell protein extracts were subjected for immunoblottings using anti‐Msi2 and Cry2. GAPDH served as an internal control. The numbers below each lane indicated the signal intensity. (*L*) Immunofluorescent staining of MyHC. MuSCs were infected by adenovirus encoding Msi2 first; virus encoding MiR7a‐1 was utilized to further infect the Msi2 overexpressing cells. The MuSCs expressing ectopic Msi2, MiR7a‐1 or both were differentiated for 48 h. The differentiated cells were subjected for immunofluorescent staining with MyHC antibody. Red indicated MyHC; blue indicated DAPI; merge indicated the merge of red and blue images. Scale bars: 100 μm. (*M*) Expression levels of MCK and MyHC. RT‐qPCR assays were performed to examine the expression levels of differentiation markers. Error bars indicated standard deviation and were based on three independent experiments. ** indicated *P* < 0.01. ns indicated no significant changes. (*N*) Scheme of Msi2 and HuR driven post‐transcriptional regulatory cascade. HuR, human antigen R; Msi2, Musashi 2; MuSCs, muscle stem cells; RT‐qPCR, quantitative reverse transcription PCR.

The function of MiR7a‐1 in myogenesis has not been characterized yet. We went on to check it. MuSCs overexpressing MiR7a‐1 displayed differentiation defects (*Figure* 4A–4C and S5A). When MuSCs were then transfected with antagomir against MiR7a‐1, differentiation was enhanced (*Figure* 4D–4F). Taken together, these results indicate that MiR7a‐1 inhibits MuSC differentiation.

Cry2 was predicted by both PicTar (https://pictar.mdc‐berlin.de/) and TargetScan(https://www.genes.mit.edu/targetscan/index.html) as a MiR7a‐1 target gene. The 3′ UTR sequences of Cry2 was cloned to the 3′ end of firefly luciferase gene driven by PGK promoter. Renilla luciferase driven by SV40 promoter in the same construct served as an internal control (*Figure* 4G). MiR7a‐1 and the construct containing the 3′ UTR of the potential target genes were co‐transfected into C2C12 myoblasts. MiR7a‐1 reduced the translation of luciferase, while mutation of the MiR7a‐1 target site relieved the translation inhibition (*Figure* 4H). These results confirmed that MiR7a‐1 targets Cry2.

Consistently, the protein levels of Cry2 decreased upon MiR7a‐1 overexpression in MuSCs (*Figure* 4I), although the mRNA was not changed (*Figure* S4A). Similarly, transfecting MuSCs with antagomir against MiR7a‐1 increased the protein levels of Cry2 (*Figure* 4I and S5B) without changing their mRNA levels (*Figure* S5C). These results confirmed the MiR7a‐1‐mediated translational repression of Cry2. Consistent with the previous results,^29^ reduction of the protein levels of Cry2 inhibited MuSC differentiation, mimicking the effects of MiR7a‐1 overexpression (*Figure* S5D–S5H). Together, these results suggest that MiR7a‐1 represses MuSCs differentiation by down‐regulating the protein levels of Cry2.

The protein level of Cry2 decreased upon Msi2 RNAi (*Figure* 4J and S5I). Similarly, overexpressing Msi2 in MuSCs led to increased protein levels of Cry2 (*Figure* 4K and S5J). Overexpression of Msi2 was able to rescue the differentiation defects caused by MiR7a‐1 and restored the expression of differentiation markers (*Figure* 4L and 4M).

Combined, these results suggest that there is a post‐transcriptional regulation cascade controlling myogenesis. Upon differentiation, RNA‐binding protein Msi2 and HuR bind *pri‐MiR7a‐1* triggering the efficient inhibition of MiR7a‐1 biogenesis to reduce the amount of mature MiR7a‐1. The reduction of mature MiR7a‐1 releases the translational inhibition of Cry2 to increase their protein levels and facilitate the differentiation of MuSCs (*Figure* 4N).

### The decline of Msi2 level attributes to ageing‐induced muscle regeneration defects

**Figure 5:**
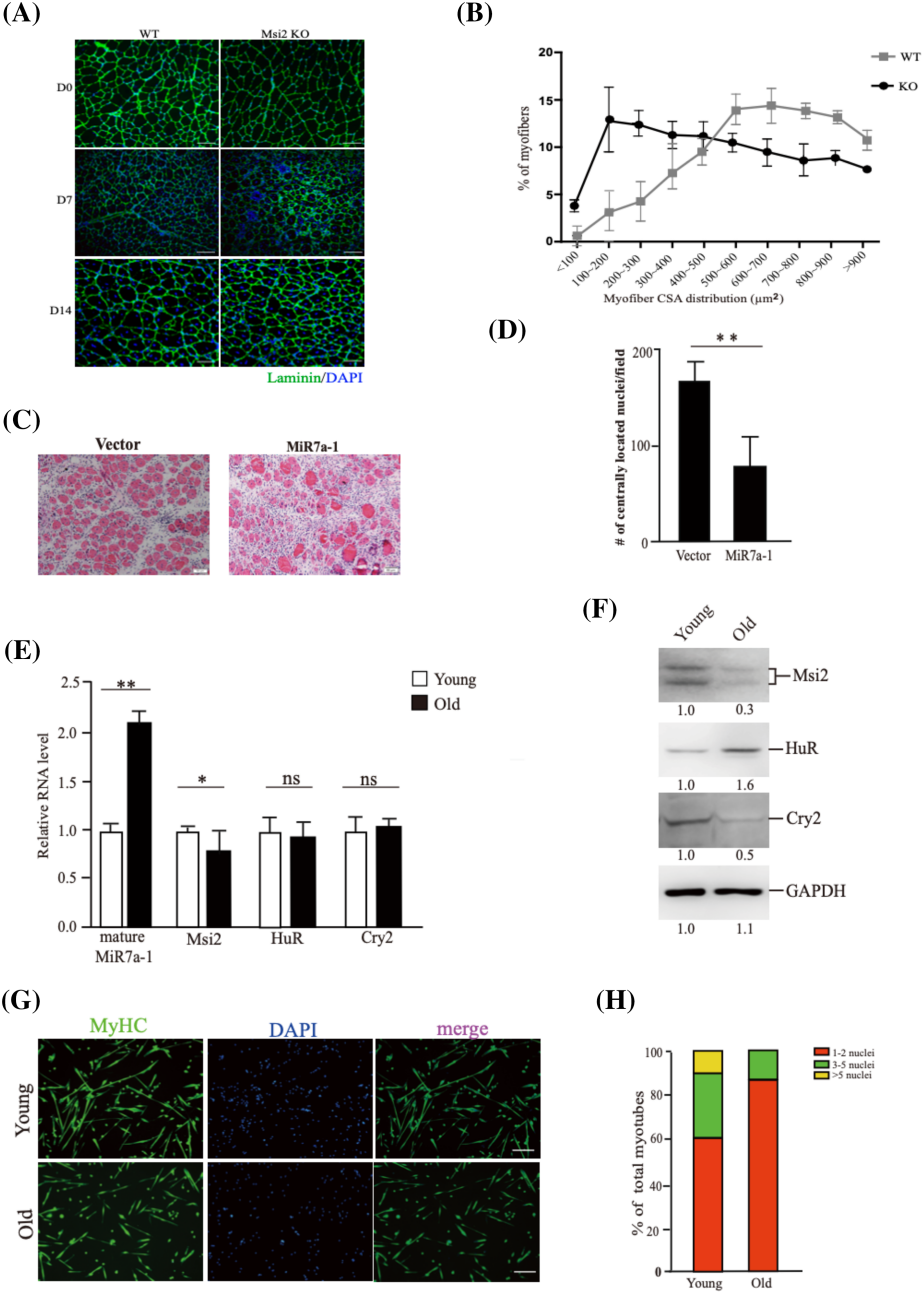
The decline of Msi2 level attributes to ageing‐induced muscle regeneration defects. (*A*) Immunofluorescence staining of Laminin and DAPI on cryosections derived from WT or Msi2 KO mice (3 months) mice on Days 7 and 14 after injury. Green indicated Laminin; blue indicated DAPI staining of nuclei; scale bars: 100 μm. (*B*) Statistical analysis of the distribution of myofibre size on Day 14 after injury. (*C*) HE staining of muscle cryosections from mice overexpressing MiR7a‐1. Adenovirus encoding MiR7a‐1 or MLP vector were injected to TA muscle intramuscularly once a day for 5 days. On the first day of injection, CTX was also injected. TA muscles were harvested 7 days after CTX injection. HE staining was performed with cryosections derived from TA muscles. Scale bars: 50 μm. (*D*) Statistical analysis of the myofibres containing centrally located nuclei. One thousand fields were counted for each mouse. Error bars indicated standard deviation and were based on five independent experiments. ** indicated *P* < 0.01. (*E*) RNA levels of mature MiR7a‐1, Msi2, HuR and Cry2 in differentiated muscle cells from young and old mice, respectively. Primary MuSCs were isolated from young (3 months) or old (24 months) TA muscles. RT‐qPCR was performed with total RNAs extracted from the differentiated cells. The results were normalized to GAPDH. Error bars indicated standard deviation and were based on three independent experiments. * indicated *P* < 0.05. ** indicated *P* < 0.01. ns indicated no significant changes. (*F*) Protein levels of Msi2, HuR and Cry2 in differentiated MuSCs isolated from young and old mice, respectively. Primary MuSCs were isolated from young (3 months) or old (24 months) TA muscles. Immunoblotting assays were performed with whole cell protein extracts from the differentiated cells. The numbers under each lane indicated the signal intensity. (*G*) Immunofluorescent staining of MyHC. MuSCs were isolated from young or old mice and differentiated for 3 days. The differentiated cells were stained with anti‐MyHC antibody. Green indicated MyHC; blue indicated DAPI staining for nuclei; merge indicated the merge of blue and green images. Scale bars: 100 μm. (*H*) Statistical analysis of the fusion index. HuR, human antigen R; KO, knockout; Msi2, Musashi 2; MuSCs, muscle stem cells; RT‐qCPT, quantitative reverse transcription PCR; WT, wild type.

To verify the function of Msi2 in myogenesis *in vivo*, we generated Msi2 KO mice (*Figure* S6A–S6C). Muscle injury was induced by CTX injection in TA muscle as reported previously. TA muscles were harvested on Days 7 and 14 after injury, respectively. The structure of TA muscle in Msi2 KO mice was more disorganized than that in WT TA muscle on Day 7 after injury as indicated by the laminin immunofluorescent staining (*Figure* 5A, D7). On Day 14, more myofibres contained centrally located nuclei compared with that in WT muscle (*Figure* S6D). The size of the regenerating muscle fibres was significantly smaller than that in WT TA muscles 7 and 14 days after injury (*Figure* 5A, 5B and S6E). The expression level of MiR7a‐1 was up‐regulated in myofibres isolated from Msi2 KO mice as indicated by RT‐qPCR assays (*Figure* S6F). The protein level of Cry2 was decreased as indicated by western blot (*Figure* S6G). These results reveal muscle regeneration defects in Msi2 KO mice and confirm the requirement of Msi2 for timely muscle regeneration.

We further checked the function of MiR7a‐1 in muscle regeneration *in vivo*. *In vivo* MiR7a‐1 overexpression was induced by injecting adenovirus encoding MiR7a‐1 or scramble sequences intramuscularly in TA muscles once a day for five continuous days as described before^30^ when muscle injury was induced by CTX injection (*Figure* S7A). The expression level of MiR7a‐1 in TA muscle was increased after injection as indicated by RT‐qPCR assays (*Figure* S7B). Cry2 protein level decreased after MiR7a‐1 overexpression (*Figure* S7C). The TA muscles overexpressing MiR7a‐1 displayed regeneration defects (*Figure* 5C and 5D & S7D and S7E). These results indicate that MiR7a‐1 inhibits muscle regeneration *in vivo*, consistent with the observations in MuSCs.

The expression of Msi2 decreased in MuSCs isolated from old mice (24 months) (*Figure* 5E and 5F), while the level of HuR remained largely unchanged. Consistently, the level of mature MiR7a‐1 increased in old MuSCs (*Figure* 5E) and the protein level of the MiR7a‐1 target gene Cry2 decreased (*Figure* 5F and S7F). Consistent with the expression level change of MiR7a‐1 and Cry2, old mice displayed muscle regeneration defects as indicated by the obviously more disorganized muscle structure and smaller myofibre size 7 days after injury (*Figure* S7G and S7H). In the same line with the above results, the differentiation ability of MuSCs isolated from old mice decreased (*Figure* 5G and 5H). These results indicate that the Msi2‐MiR7a‐1‐Cry2 axis of post‐transcriptional cascade is disrupted during skeletal muscle ageing, which could attribute to the muscle regeneration defect of aged muscles.

## Discussion

Post‐transcriptional regulation is a key step to regulate many cellular processes. Here, we found that a post‐translational cascade governed by a pair of RNA‐binding protein Msi2 and HuR is required for muscle regeneration. Msi2 and HuR up‐regulates the protein level of Cry2 by repressing the biogenesis of MiR7a‐1. This cascade is disrupted during ageing, which attributes to the decline of muscle regeneration ability in old individuals. These findings identify a new post‐transcriptional cascade in myogenesis and reveal its important functions in skeletal muscle ageing, which provides new targets to improve the muscle regeneration ability in aged skeletal muscles.

Both Msi2 and HuR have been reported to promote oncogenesis and metastasis in many tissues such as blood, lung, breast, liver, pancreas and bladder.^14^, ^31^, ^32^, ^33^, ^34^, ^35^, ^36^ Msi2 and HuR negatively regulate the maturation of MiR7a‐1.^28^ MiR‐7 has been shown to inhibit the proliferation, migration and invasion of colorectal cancer, breast cancer, papillary cancer and many other types of cancer cells,^37^, ^38^, ^39^, ^40^, ^41^ which agrees with the tumorigenic roles of Msi2 and HuR. Consistent with their roles as tumour drivers, Msi2 and HuR are strong promoters of stem cell proliferation and inhibitor of differentiation.^42^, ^43^ However, both Msi2 and HuR function in the opposite direction in muscle cells. Our results indicate that muscle cells rewire the RNA‐binding protein cascade and use it to promote MuSC differentiation. This may partially contribute to the oncogenic resistant feature of skeletal muscle.

Many reports suggest that mRNA levels do not necessarily correlate with protein levels.^44^, ^45^, ^46^ Here, we found that the protein level of HuR and Cry2 changed in old MuSCs, while their mRNA levels were kept unchanged. It highly suggests that post‐transcriptional regulation is an important player in ageing. When cell status changes, the cellular responses could be achieved faster in the form of post‐transcriptional regulation.^44^, ^47^ The transcriptional regulation of MiRs during myogenesis has been well studied.^48^, ^49^ Our results reveal that regulation of microRNA biogenesis also plays critical roles in myogenesis. Although HuR and Msi2 are ubiquitously expressed in many cell types, several observations seem to suggest that the binding site choice made by these two factors is cell context dependent.^28^ How the specificity is achieved is an interesting question for further exploration.

## Conflict of interest

The authors declare no conflicts of interests.

## Funding

This work was supported by the Strategic Priority Research Program of the Chinese Academy of Science (XDA16020400 to P.H.), Ministry of Science and Technology of China (2017YFA0102700 to P.H.), the National Natural Science Foundation of China (32170804 to P.H. and 81200355 to W.Y.), CAS‐Youth Innovation Program Association (2016246 to W.Y.), Shanghai Natural Science Foundation (18ZR1446300 to W.Y.).

## Supporting information


**Figure S1.** Msi1 expression decreased in differentiated MuSCs. (Related to Figure 1). Expression levels of Msi1 in primary MuSCs before and after differentiation, respectively. RT‐qPCR was performed with RNA extracted from the primary MuSCs before or after differentiation. The results were normalized to GAPDH. Error bars indicated standard deviation and were based on 3 independent experiments. ***indicated *p* < 0.001.
**Figure S2.** HuR is required for myogenesis. (Related to Figure 2).
**Figure S3.** Msi2 is required for efficient MiR7a‐1 processing. (Related to Figure 2).
**Figure S4.** Msi2 and Hur work cooperatively to repress the processing of MiR7a‐1. (Related to Figure 2).
**Figure S5.** MiR7a‐1 targets Cry2. (Related to Figure 4).
**Figure S6.** Characterization of Msi2 KO muscle after CTX injury. (Related to Figure 5).
**Figure S7.** Over‐expression of MiR7a‐1 in TA muscle leads to muscle regeneration defects. (Related to Figure 5).
