# Supplementary material for: Msi2‐mediated MiR7a‐1 processing repression promotes myogenesis
Source: J Cachexia Sarcopenia Muscle. 2021 Dec 8;13(1):728–42. doi: 10.1002/jcsm.12882 (PMC8818652; doi:10.1002/jcsm.12882)
Supplement: Supplementary file 1 — Figure S1. Msi1 expression decreased in differentiated MuSCs. (Related to Figure 1). Expression levels of Msi1 in primary MuSCs before and after differentiation, respectively. RT‐qPCR was performed with RNA extracted from the primary MuSCs before or after differentiation. The results were normalized to GAPDH. Error bars indicated standard deviation and were based on 3 independent experiments. ***indicated p < 0.001. Figure S2. HuR is required for myogenesis. (Related to Figure 2). Figure S3. Msi2 is required for efficient MiR7a‐1 processing. (Related to Figure 2). Figure S4. Msi2 and Hur work cooperatively to repress the processing of MiR7a‐1. (Related to Figure 2). Figure S5. MiR7a‐1 targets Cry2. (Related to Figure 4). Figure S6. Characterization of Msi2 KO muscle after CTX injury. (Related to Figure 5). Figure S7. Over‐expression of MiR7a‐1 in TA muscle leads to muscle regeneration defects. (Related to Figure 5). [file JCSM-13-728-s001.pdf]

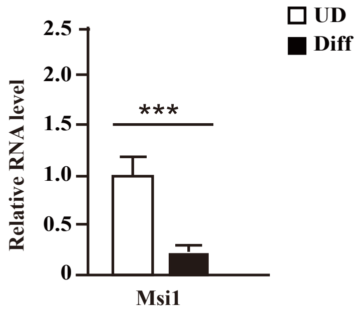

Supplemental Figure 1. Msi1 expression decreased in differentiated MuSCs. (Related to Figure 1) Expression levels of Msi1 in primary MuSCs before and after differentiation, respectively. RT-qPCR was performed with RNA extracted from the primary MuSCs before or after differentiation. The results were normalized to GAPDH. Error bars indicated standard deviation and were based on 3 independent experiments. ***indicated p<0.001.

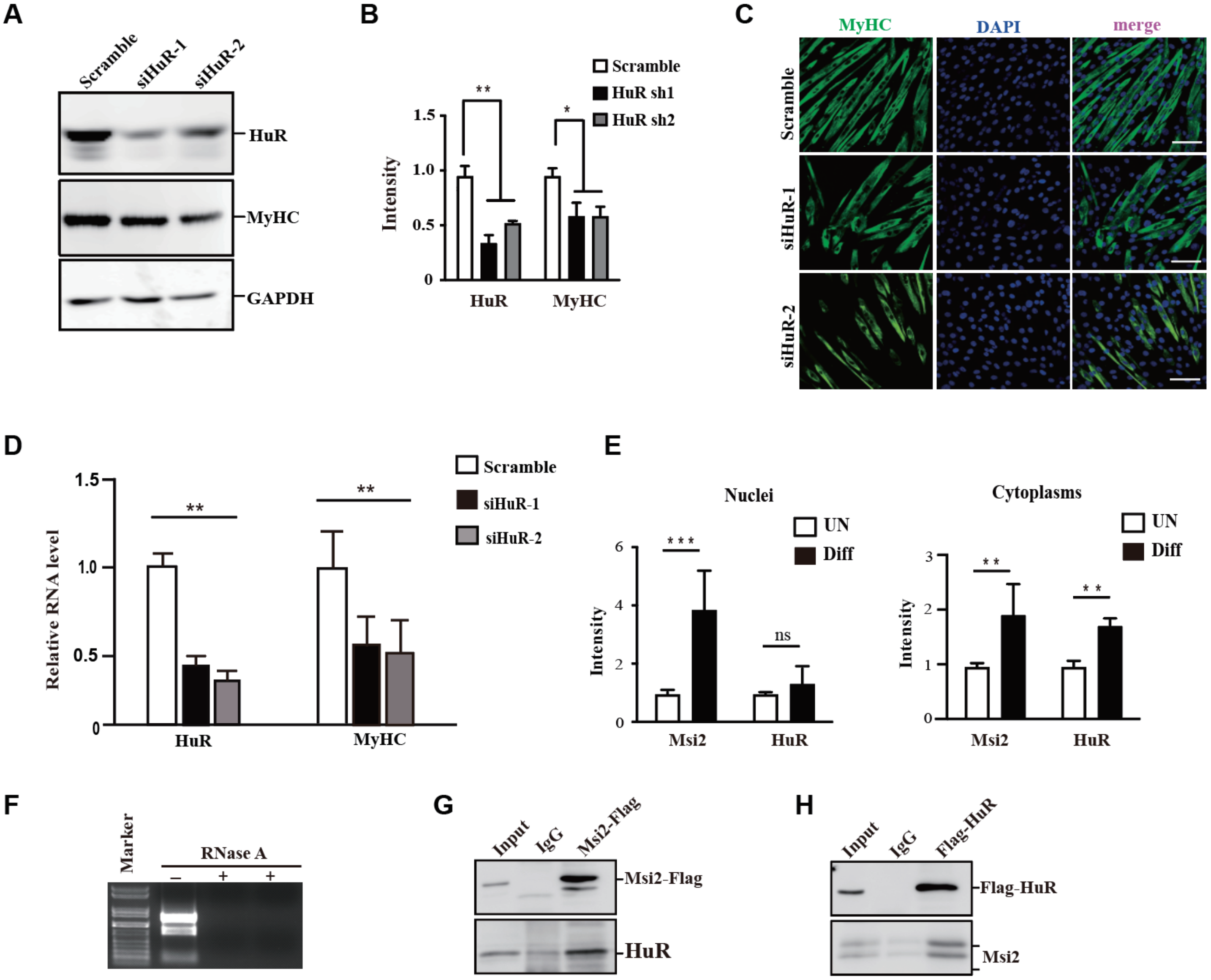

Supplemental Figure 2. HuR is required for myogenesis. (Related to Figure 2)

A. Immunoblotting of HuR after RNAi. Two pieces of siRNA against HuR were transfected to MuSCs, respectively. The transfected MuSCs were differentiated for 3 days. The whole cell protein extracts from the transfected cells were subjected for immunoblotting with antibodies against HuR, MyHC, and GAPDH. MyHC marked the differentiation status. GAPDH served as internal control.

B. Quantification of Western blots shown in SFig. 2A. Error bars indicated standard deviation and were based on 3 independent experiments. * indicated $p<0.05$. ** indicated $p<0.01$.

C. Immunofluorescent staining of MyHC in differentiated MuSCs after RNAi. Two pieces of siRNA against HuR were transfected to MuSCs. The transfected MuSCs were differentiated for 3 days followed by immunofluorescent staining with anti-MyHC antibody. Green indicated MyHC; DAPI indicated nuclear staining; merge indicated the merge of green and blue images. Scale bars: 50µm.

D. The expression levels of HuR and MyHC. MuSCs were transfected with 2 pieces of siRNA against HuR, respectively. The transfected MuSCs were differentiated for 3 days. RT-qPCR assays were performed with total RNA extracted from the differentiated cells. The results were normalized to GAPDH. Error bars indicated standard deviation and were based on 3 independent experiments. ** indicated $p<0.01$.

E. Quantification of Western blots shown in Fig. 2D. Error bars indicated standard deviation and were based on 3 independent experiments. ** indicated $p<0.01$. *** indicated $p<0.001$. ns indicated no significant change.

F. Protein extracts from MuSCs were treated with RNase A and subjected for agarose gel electrophoresis.

G. Immunoprecipitation followed by immunoblotting to confirm the protein-protein interaction between Msi2 and HuR. MuSCs were infected by adenovirus encoding Flag-tagged Msi2. Whole cell protein extracts from the infected cells were subjected to anti-Flag immunoprecipitation followed by HuR immunoblotting. IgG immunoprecipitation serves as the control.

H. Immunoprecipitation followed by immunoblotting to confirm the protein-protein interaction between Msi2 and HuR. MuSCs were infected by adenovirus encoding Flag-tagged HuR. Whole cell protein extracts from the infected cells were subjected to anti-Flag immunoprecipitation followed by Msi2 immunoblotting. IgG immunoprecipitation serves as the control.

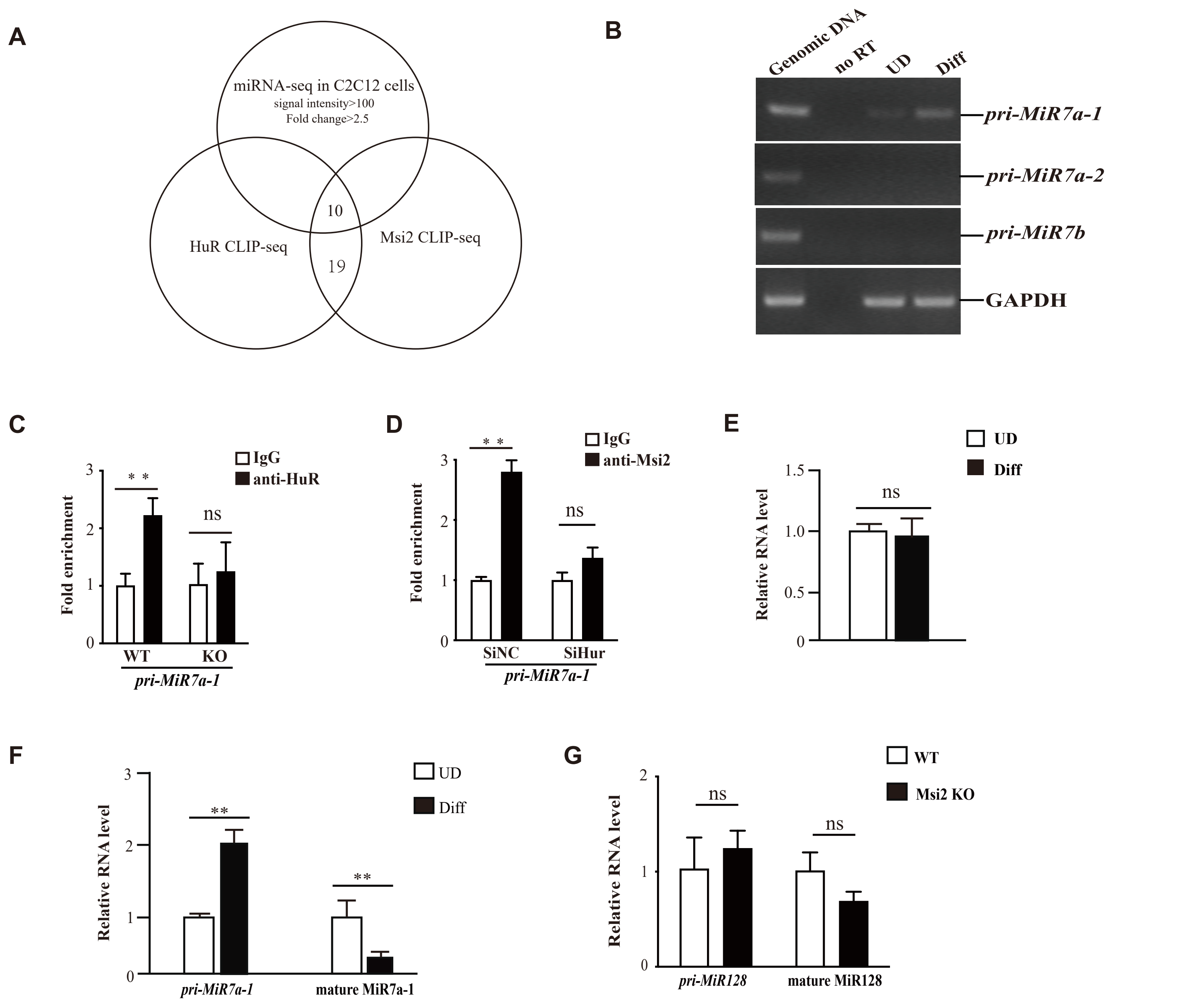

Supplemental Figure 3. Msi2 is required for efficient MiR7a-1 processing. (Related to Figure 2)

A. The overlapping of mRNA-seq of C2C12, HuR CLIP-seq, and Msi2 CLIP-seq data.

B. Semi-quantitative RT-PCR of *pri-MiR7* isoforms. Semi-quantitative RT-PCR assays were performed with primers specific for *pri-MiR7a-1*, *pri-MiR7a-2*, and *pri-MiR7b*. GAPDH served as the internal control.

C. RIP-PCR assays using anti-HuR in differentiated Msi2 KO MuSCs. Error bars indicated standard deviation and were based on 3 independent experiments. ** indicated $p<0.01$. ns indicated no significant changes.

D. RIP-PCR assays using anti-Msi2 in differentiated MuSCs treated with HuR siRNA. Error bars indicated standard deviation and were based on 3 independent experiments. ** indicated $p<0.01$. ns indicated no significant changes.

E. The expression level of *Hnrnpk* in undifferentiated and differentiated MuSCs. MuSCs were differentiated for 3 days. RT-qPCR assays were performed with the total RNA extracted from MuSCs before or after differentiation. Error bars indicated standard deviation and were based on 3 independent experiments. ns indicated no significant changes.

F. The expression level of *pri-MiR7a-1* and mature MiR7a-1 in undifferentiated and differentiated MuSCs. RT-qPCR assays were performed with the total RNA extracted from MuSCs before or after differentiation. Error bars indicated standard deviation and were based on 3 independent experiments. ** indicated $p<0.01$.

G. The expression level of *pri-MiR128* and mature MiR128 in WT and Msi2 KO MuSCs. MuSCs were isolated from WT and Msi2 KO mice followed by differentiation. RT-qPCR assays were performed using total RNAs extracted from the differentiated cells. Error bars indicated standard deviation and were based on 3 independent experiments. ns indicated no significant changes.

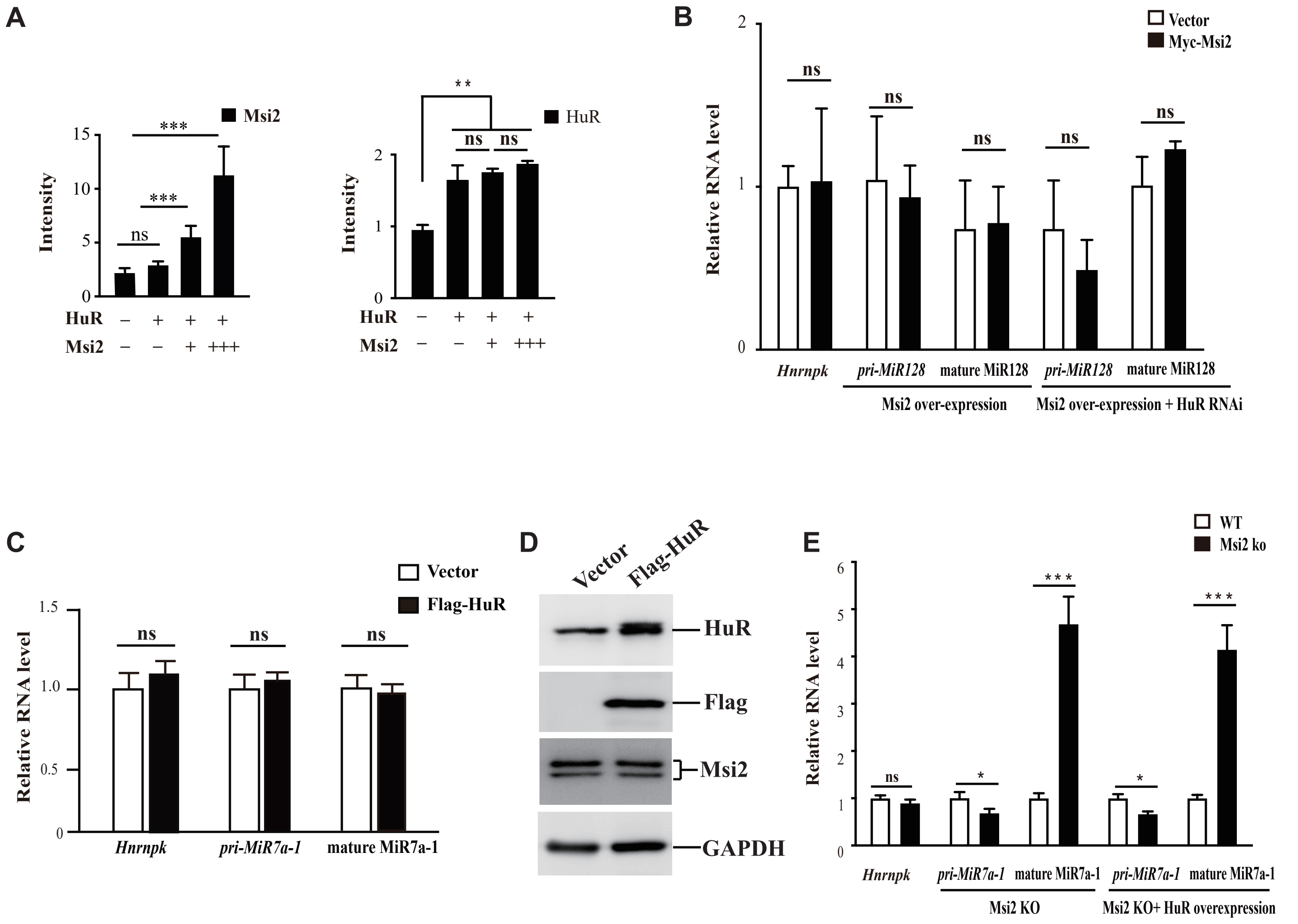

Supplemental Figure 4. Msi2 and Hur work cooperatively to repress the processing of MiR7a-1. (Related to Figure 2)

A. Quantification of Western blots shown in Fig. 2L. Error bars indicated standard deviation and were based on 3 independent experiments. ** indicated $p<0.01$. *** indicated $p<0.001$. ns indicated no significant changes.

B. The expression levels of *Hnrnpk, pri-MiR128*, and mature MiR128 in MuSCs ectopically expressing Myc-tagged Msi2. RT-qPCR assays were performed to detect the expression levels of *Hnrnpk, pri-MiR128*, and mature MiR128. ShRNA against HuR was further introduced to MuSCs over-expressing Msi2. The expression levels of *pri-MiR128* and mature MiR128 were examined by RT-qPCR assays. Error bars indicated standard deviation and were based on 3 independent experiments. ns indicated no significant changes.

C. The expression levels of *pri-MiR7a-1*, mature MiR7a-1, and *Hnrnpk* in MuSCs overexpressing HuR. MuSCs were infected by adenovirus encoding Flag tagged HuR. RT-qPCR assays were performed with the total RNA. Error bars indicated standard deviation and were based on 3 independent experiments. ns indicated no significant changes.

D. The protein levels of HuR and Msi2 in MuSCs overexpressing HuR. MuSCs were infected by adenovirus encoding Flag tagged HuR. Immunoblotting assays were performed with the whole cell protein extracts. GAPDH served as the internal control.

E. The levels of *pri-MiR7a-1* and mature MiR7a-1in differentiated Msi2 KO MuSCs or differentiated Msi2 KO MuSCs over-expressing HuR were examined by RT-qPCR. Error bars indicated standard deviation and were based on 3 independent experiments. * indicated $p<0.05$. *** indicated $p<0.001$. ns indicated no significant changes.

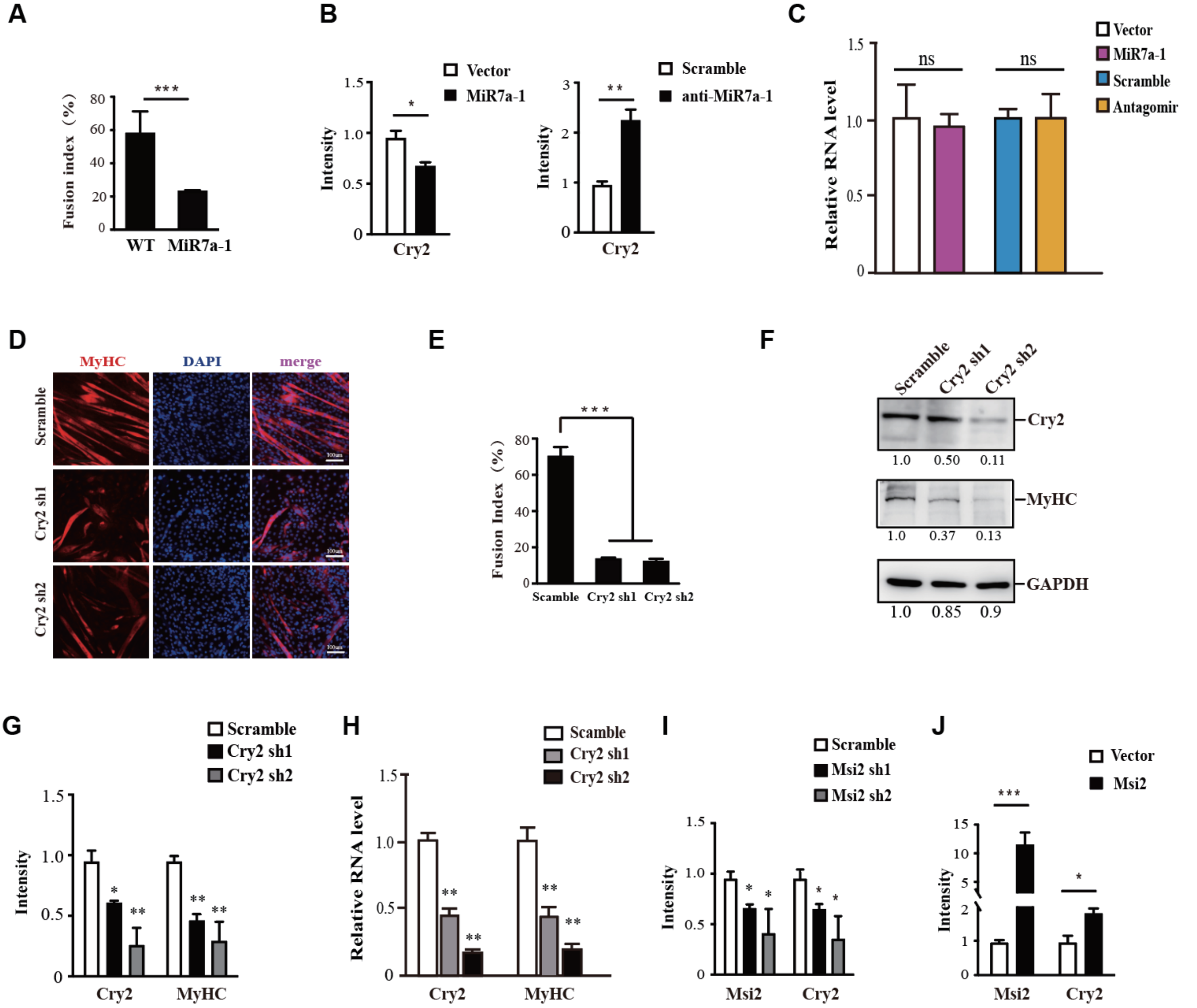

Supplemental Figure 5. MiR7a-1 targets Cry2. (Related to Figure 4)

A. Quantification of fusion index in Fig. 4A. Error bars indicated standard deviation and were based on 3 independent experiments. *** indicated p<0.001.

B. Quantification of Western blots shown in Fig. 4I. Error bars indicated standard deviation and were based on 3 independent experiments. * indicated p<0.05. ** indicated p<0.01.

C. The expression levels of Cry2 in MuSCs over-expressing MiR7a-1 or antagomir against MiR7a-1. RT-qPCR assays were performed with total RNA extracted from MuSCs over-expressing MiR7a-1 or antagomir against MiR7a-1. Error bars indicated standard deviation and were based on 3 independent experiments. ns indicated no significant changes.

D. Immunofluorescent staining of MyHC in Cry2 RNAi MuSCs. MuSCs were infected by retrovirus encoding shRNA against Cry2 and then differentiated. Two pieces of shRNA were utilized. Red indicated MyHC; DAPI indicated nuclear staining; merge indicated merged images of red and blue. Scale bars: 100μm.

E. Quantification of fusion index in sFig. 5D. Error bars indicated standard deviation and were based on 3 independent experiments. *** indicated p<0.001.

F. Protein level of Cry2 and MyHC after RNAi. MuSCs were infected by retrovirus encoding shRNA against Cry2 and then differentiated. Two pieces of shRNA were utilized. The whole cell protein extracts were subjected for immunoblotting. GAPDH served as the internal control.

G. Quantification of Western blots shown in sFig. 5F. Error bars indicated standard deviation and were based on 3 independent experiments. * indicated p<0.05. ** indicated p<0.01.

H. Expression levels of MyHC and Cry2 in Cry2 RNAi cells. MuSCs were infected by retrovirus encoding shRNA against Cry2 and then differentiated. Two pieces of shRNA were utilized. RT-qPCR assays were performed with the total RNA. Error bars indicated standard deviation and were based on 3 independent experiments. ** indicated p<0.01.

I. Quantification of Western blots shown in Fig. 4J. Error bars indicated standard deviation and were based on 3 independent experiments. * indicated p<0.05.

J. Quantification of Western blots shown in Fig. 4K. Error bars indicated standard deviation and were based on 3 independent experiments. * indicated p<0.05. *** indicated p<0.001.

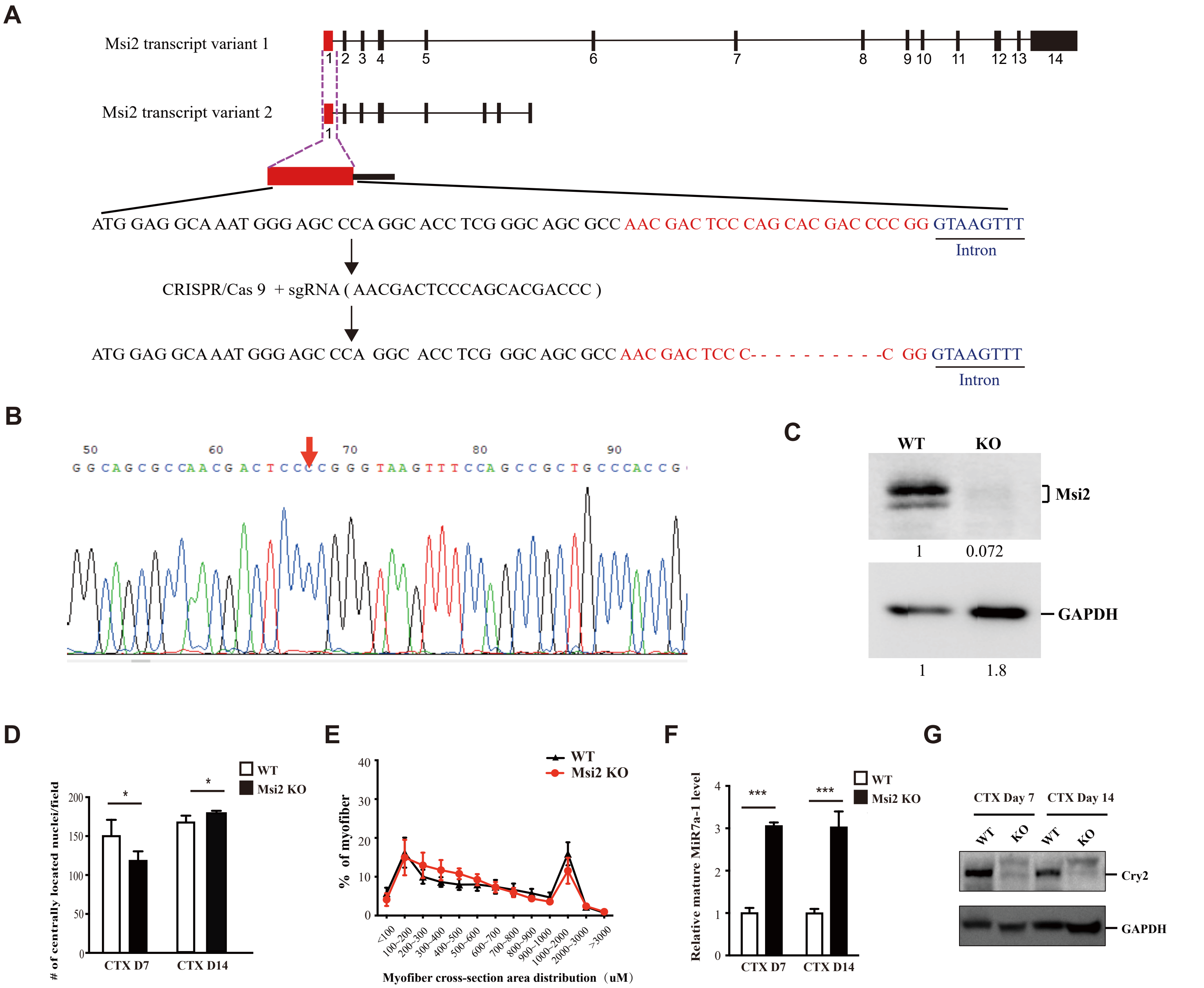

Supplemental Figure 6. Characterization of Msi2 KO muscle after CTX injury. (Related to Figure 5)

A. The scheme to generate Msi2 KO mice.

B. DNA sequence of the starting nucleotides of the frame shift.

C. Immunoblotting of Msi2 in WT and Msi2 KO mice. The whole cell protein extracts from skeletal muscles of WT or Msi2 KO mice were subjected for immunoblotting assays using antibodies against Msi2 and GAPDH. Gapdh served as an internal control. The numbers below each panel indicated relative signal intensity.

D. Quantification of the number of myofibers containing centrally located nuclei day 7 and day 14 post CTX injury. Error bars indicated standard error based on 5 independent experiments. * indicated $p<0.05$.

E. Statistical analysis of the myofiber size distribution day 7 post CTX injury.

F. Expression level of MiR7a-1 measured by RT-qPCR at day 7 and day 14 post CTX injury. Error bars indicted standard error based on 5 independent experiments. *** indicated $p<0.001$.

G. Protein level of Cry2 measured by Western blot at day 7 and day 14 post CTX injury.

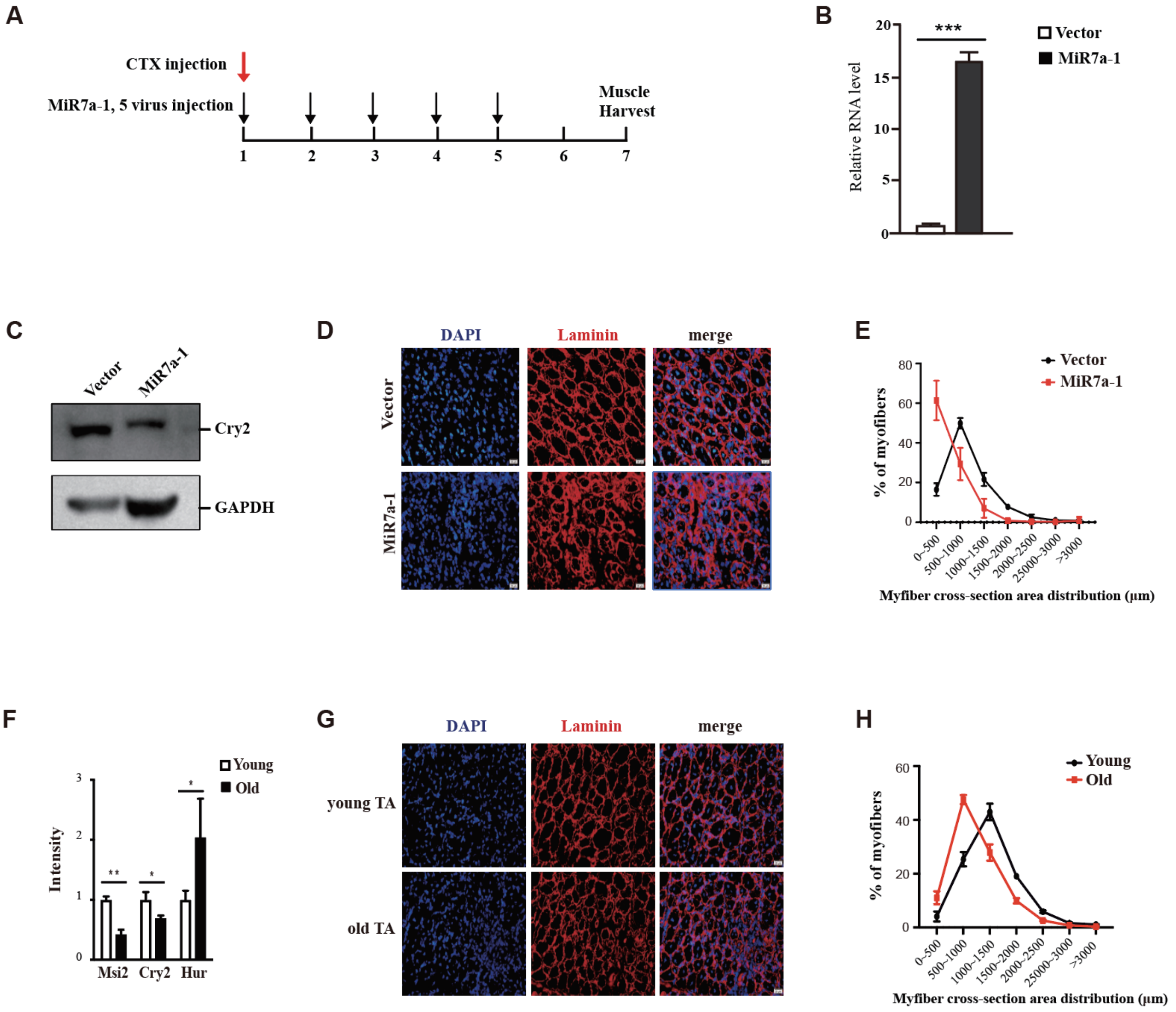

Supplemental Figure 7. Over-expression of MiR7a-1 in TA muscle leads to muscle regeneration defects. (Related to Figure 5)

A. The scheme of MiR7a-1 virus and CTX injection.

B. The expression level of MiR7a-1 in TA muscle infected by adenovirus encoding MiR7a-1. Adenovirus was injected into TA muscle intramuscularly once a day for 5 continuous days. TA muscle was harvested at day 7 (2 days after the last injection). RT-qPCR assays were performed with the total RNA extracted from TA muscle. Error bars indicated standard deviation and were based on 3 independent experiments. *** indicated $p<0.001$.

C. Protein level of Cry2 in TA muscles over-expressing MiR7a-1 as indicated by Western blot.

D. Immunofluorescence staining of Laminin and DAPI on cryosections derived from TA muscles injected with adenovirus encoding either vector or MiR7a-1. Red indicated Laminin; DAPI indicated the staining of nuclei; merge indicated the merged images of Laminin and DAPI. Scale bars: 20μm.

E. Statistical analysis of the myofiber size of TA muscles expressing vector or MiR7a-1 on day 7 post injury.

F. Quantification of Western blots shown in Fig. 5F. Error bars indicated standard deviation and were based on 3 independent experiments. * indicated $p<0.05$. ** indicated $p<0.01$.

G. Old mice display muscle regeneration defects. Immunofluorescent staining of Laminin and DAPI on cryosections derived from young (3 months) or old (20 months) mice on day 7 post injury. Red indicated Laminin staining; DAPI indicated staining of nuclei; merge indicated the merged images of Laminin and DAPI staining. Scale bars: 20μm.

H. Statistical analysis of myofiber size of old or young TA on day 7 post injury.
